# Multi-scale biomimetic fusion construction of cerium ion hydrogel-scaffold for promoting osteoporotic bone defect repair

**DOI:** 10.1016/j.jot.2025.08.015

**Published:** 2025-09-13

**Authors:** Yesheng Jin, Shuqing Lv, Nanning Lv, Yixue Huang, Jia Wang, Yun Xiao, Xinfeng Zhou, Yanxia Ma, Gang Zhao, Fan He, Yong Xu

**Affiliations:** a Department of Orthopedic, The First Affiliated Hospital of Soochow University, MOE Key Laboratory of Geriatric Diseases and Immunology, Suzhou Medical College, Soochow University, Suzhou, 215000, Jiangsu, China; b MOE Key Laboratory of Geriatric Diseases and Immunology, School of Life Science, Suzhou Medical College, Soochow University, Suzhou, 215000, China; c Department of Orthopedic Surgery, Lianyungang Clinical College, Xuzhou Medical University & The Second People's Hospital of Lianyungang, 41 Hailian East Street, Lianyungang, Jiangsu, 222003, China; d Department of Orthopedic, Soochow University Affiliated Wuxi Ninth People's Hospital, Wuxi, 214061, China

**Keywords:** Alendronate, Bone regeneration, Calcium ion transport, Cerium, Osteoporotic bone defect, Wnt pathway

## Abstract

**Background:**

The treatment of bone defects in the context of osteoporosis encounters numerous challenges. In the osteoporotic microenvironment, bone resorption outweighs bone formation, impeding the self-repair of bone defect areas. Furthermore, the deterioration of osteogenesis-angiogenesis coupling function at the defect sites and excessive inflammatory responses further complicate the treatment of bone defects. Hence, an improved approach is urgently needed to enhance the treatment of osteoporotic bone defects.

**Methods:**

Our efficient strategy has developed a multi-scale biomimetic fusion alendronate sodium cerium ion hydrogel scaffold, integrating 3D-printed tricalcium phosphate (TCP) scaffolds, collagen-methacrylate (COMA) hydrogel, and nanoparticles of alendronate sodium cerium ions. *In vitro*, we intervened osteoporosis rat derived bone marrow stromal cells (BMSCs) with the extract of TCP-H-Alendronate sodium cerium ion nanoparticles (ACNP) scaffold and detected the osteogenesis-related indicators through alkaline phosphatase (ALP) enzymatic activity staining, alizarin red staining, Western Blot, RT-qPCR and immunofluorescence staining to evaluate the osteogenic differentiation effect of TCP-H-ACNP scaffold. Through transcriptome sequencing, we explored the mechanism of TCP-H-ACNP scaffold affecting osteogenic differentiation of osteoporotic BMSCs. We intervened human umbilical vein endothelial cells (HUVECs) with the extract of TCP-H-ACNP scaffold and evaluated the angiogenic effect of TCP-H-ACNP scaffold through tube formation assay and cell scratch assay. *In vivo*, we established a distal femoral bone defect model in osteoporotic rats and evaluated the therapeutic effect *in vivo* through Mirco CT, Hematoxylin and Eosin (H&E) stainin, Masson staining and immunohistochemical staining.

**Results:**

The results demonstrated that *invitro*, TCP-H-ACNP scaffolds could promote osteogenic differentiation of osteoporotic BMSCs from rats and angiogenesis of HUVECs. *In vivo*, TCP-H-ACNP scaffolds could promote bone regeneration and repair of distal femoral bone defects in osteoporotic rats and improve local angiogenesis. Mechanistically, TCP-H-ACNP scaffolds could directly promote osteogenic differentiation of osteoporotic BMSCs from rats through the Wnt signaling pathway, and indirectly promote osteogenic differentiation by influencing Ca ion transport and improving mitochondrial function.

**Conclusion:**

We create a hydrogel scaffold that not only offers adequate mechanical support but also possesses a favorable microenvironment for cell growth and contains biological factors promoting osteogenic and angiogenic differentiation.

**The translational potential of this paper:**

This application represents a pioneering aspect of multi-scale biomimetic hydrogel scaffolds in addressing osteoporotic bone defects, providing a novel direction for the treatment of osteoporotic bone defects.

## Graphical abstract

**Figure:**
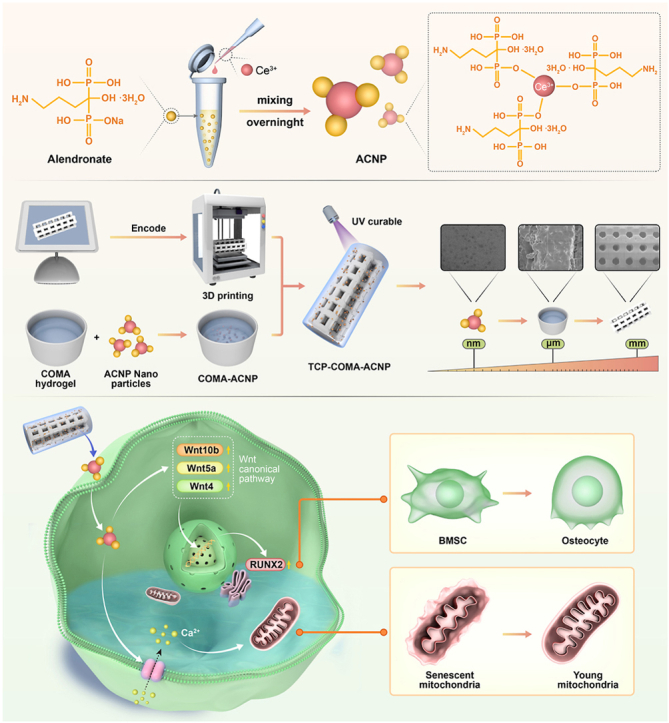

## 1 Introduction

Bone defect treatment has always been a difficult and hot topic in orthopedic disease treatment. Its causes include trauma, inflammation, tumors, etc. With the gradual aging of the population, the incidence of osteoporotic bone defects is increasing [1]. It is characterized by high disability and mortality rates and a heavy economic burden. For patients with osteoporotic bone defects, they not only face the difficulties of conventional bone defect treatment, but also the osteoporotic microenvironment at the defect site further increases the difficulty of bone defect treatment. In the osteoporotic microenvironment, bone destruction is greater than bone formation, which hinders the self-repair of the bone defect site. Moreover, the decline in osteogenesis-angiogenesis coupling function at the defect site and excessive inflammatory responses further increase the difficulty of bone defect treatment [2,3].

Alendronate sodium (Aln), a drug approved by the US Food and Drug Administration (FDA) for osteoporosis treatment, has shown the ability to suppress the regeneration of osteoclasts [4,5]. Nevertheless, when applied in the repair of bone defects, it fails to adhere to the bone at the defect site, thereby being unable to exert a bone repair effect; its limited efficacy in improving blood supply and the inflammatory microenvironment at the defect site restricts its further advancement in the biological domain [6,7]. The self-assembly of supramolecular materials, mediated by metal–ligand coordination, has emerged as a powerful strategy for enhancing ligand bioavailability [8]. Unlike conventional carrier-dependent delivery systems, this approach circumvents critical limitations such as suboptimal drug loading capacity, formulation instability, and carrier-induced biocompatibility concerns. Importantly, these metal-coordinated supramolecular architectures exhibit stimuli-responsive behavior through reversible coordination bond dissociation, enabling dynamic size modulation. These unique properties position metal-alendronate sodium supramolecular complexes as particularly promising candidates for therapeutic intervention in osteoporotic bone regeneration.

Bioactive ion doping has emerged as a promising strategy for functionalizing bone repair materials. This approach enables the enhancement of inherent physical, chemical, and biological properties of biomaterials while simultaneously delivering essential trace elements to support bone metabolism. Notably, numerous studies have confirmed that incorporating therapeutic ions such as magnesium (Mg^2+^), zinc (Zn^2+^), and strontium (Sr^2+^) into scaffolds significantly improves their osteogenic and angiogenic capacity *in vivo*. Of particular interest is cerium (Ce^3+^/^4+^), a rare earth element with demonstrated therapeutic potential for various bone disorders. Physiological evidence reveals substantial cerium deposition in human bone tissue, suggesting skeletal tropism and potential accumulation mechanisms. These findings highlight cerium-doped biomaterials as a compelling avenue for developing next-generation bone grafts [9]. Furthermore, due to their similar ionic radii, they can readily substitute for some of the Ca^2+^ ions in the apatite nanoparticles [10]. Within the safe concentration range, the element cerium (Ce) is employed as a therapeutic agent to induce the growth of bone tissue by enhancing angiogenic and osteogenic activities [11,12]. Recent studies in bone tissue engineering have highlighted the significant influence of cerium on osteogenic activity. As the primary cells responsible for bone matrix production and mineralization, osteoblasts exhibit enhanced functionality when exposed to cerium ions. Mechanistically, Ce ions stimulate osteogenesis through diverse biological pathways. First, they induce the differentiation of bone marrow stromal cells (BMSCs) into osteoblasts and accelerate mineral deposition by upregulating the Wnt/β-catenin cascade [13]. Furthermore, Ce ions contribute to extracellular matrix remodeling by boosting collagen production, thereby improving bone biomechanical strength [14]. At the molecular level, these ions upregulate key osteogenic markers, including osteocalcin (OCN) and collagen type I (Col I), which are critical for bone formation and structural integrity [15]. Notably, cerium ions demonstrate catalase- and superoxide dismutase–mimetic activities, enabling them to effectively scavenge reactive oxygen species (ROS). Through these dual enzymatic mechanisms, Ce^3+^/^4+^ ions can mitigate oxidative stress in osteoporotic bone tissue by catalyzing the conversion of superoxide radicals (O_2_^-^) to hydrogen peroxide (H_2_O_2_) and subsequently to water (H_2_O) and oxygen (O_2_) [5,16]. In this experiment, we replaced three sodium ions in alendronate sodium with Ce ions to form new nanoparticles. Through the modification of alendronate sodium, the newly obtained nanoparticles possess the functions of both alendronate sodium and Ce ions, achieving complementary advantages. In the early stage, Ce ions improve the microenvironment of osteoporosis and promote osteogenesis and angiogenesis. In the later stage, alendronate sodium exerts its anti-osteoporosis effect. alliance between giants.

Bone tissue mainly comprises organic and inorganic portions. The organic portion is constituted by collagen fibers and matrix, the latter mainly encompassing glycoproteins and proteoglycans, which possess certain elasticity and tenacity, and provide a favorable extracellular environment for cells. The inorganic portion mainly consists of hydroxyapatite crystals, neutral salts rich in calcium and phosphorus, mainly playing a role of mechanical support [[17], [18], [19]]. Based on the mineral components of bones, for the scaffold materials of bone grafts, the currently prevailing ones are still calcium and phosphate minerals, such as hydroxyapatite [20], tricalcium phosphate [21,22], calcium sulfate [23], and bioactive glass [24], etc. Among these materials, tricalcium phosphate is favored by all due to its excellent biocompatibility, mechanical properties, and easier absorption and degradation in the body along with the formation of new bone. Nevertheless, when it comes to repairing osteoporotic bone defects, it still confronts disadvantages like insufficient osteoinductivity, poor angiogenesis, and weak immune and anti-inflammatory capabilities. The biological performances of alendronate-cerium ion nanoparticles can also compensate for these deficiencies.

The natural extracellular matrix (ECM) has been widely employed as a biological scaffold to facilitate stem cell adhesion, migration, differentiation, and proliferation. However, its clinical translation has been limited by inherent drawbacks, including suboptimal mechanical strength and uncontrolled degradation kinetics. These limitations have prompted the development of synthetic scaffold alternatives with tunable physicochemical properties for improved regenerative outcomes [25]. Hydrogels have emerged as highly promising biomaterials owing to their exceptional water retention capacity and structural similarity to native soft tissues. Various fabrication strategies have been developed to engineer biomimetic hydrogels with tunable biophysical and biochemical properties that can precisely direct cellular differentiation processes. Furthermore, hydrogels exhibit excellent drug loading efficiency and controllable release kinetics. When combined with nanoparticles, these hybrid systems enable effective nanoparticle encapsulation and sustained release, thereby prolonging their therapeutic efficacy [26,27].

The Wnt signaling cascade serves as a critical regulator of osteogenic differentiation, comprising two principal branches: the canonical (β-catenin-dependent) and non-canonical (β-catenin-independent) pathways, both essential for skeletal development. Through the canonical pathway, Wnt signaling exerts dual osteogenic effects: (1) transcriptional upregulation of osteogenic master regulators, including RUNX2 and Osterix, and (2) inhibition of osteoclastogenesis via osteoblast-derived osteoprotegerin (OPG) production [28,29]. The non-canonical Wnt pathway is also of vital significance to osteoblasts. The β-catenin-independent Wnt pathway modulates osteogenic differentiation through calcium-mediated mechanisms, involving intracellular Ca^2+^ accumulation and subsequent activation of both calmodulin and calcineurin signaling cascades [30,31].

Mitochondria play an essential role in maintaining cellular energy metabolism, redox balance, and calcium homeostasis in BMSCs—all of which are critical for successful osteogenic differentiation. Mitochondrial dysfunction has been linked to impaired stem cell viability, increased oxidative stress, and defective lineage commitment, highlighting its importance in the regenerative capacity of bone tissue. A substantial amount of energy is consumed during the process of osteogenic differentiation to enable osteoblasts to synthesize a large number of collagens and matrix proteins. The uptake of mitochondrial Ca^2+^ activates the tricarboxylic acid (TCA) cycle and the electron transport chain (ETC), thus rapidly enhancing ATP production for osteogenic differentiation [31,32].

**Scheme 1:**
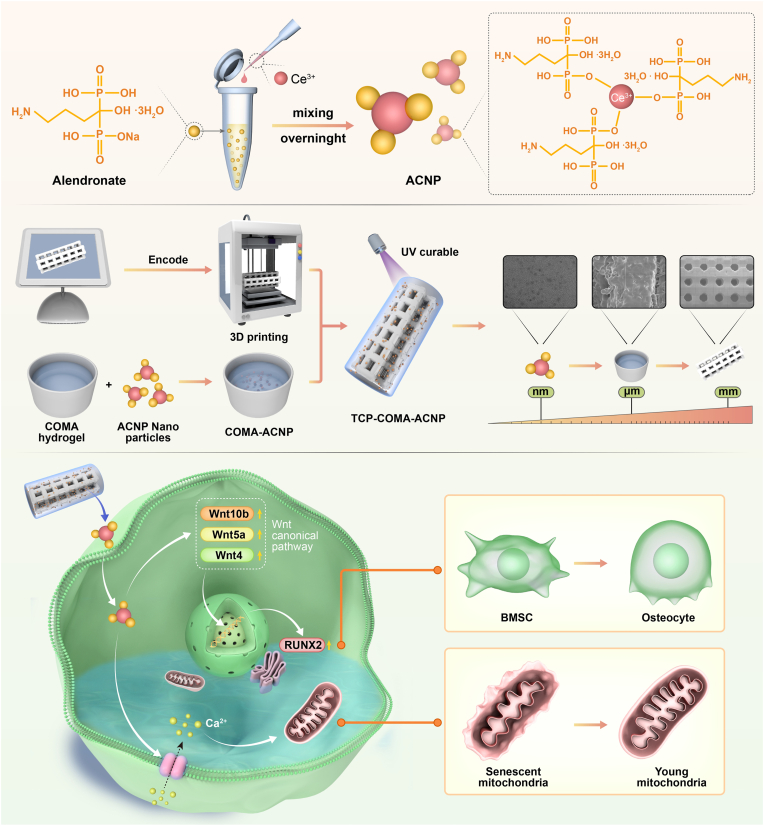
Schematic diagram illustrating the mechanism of TCP-H-ACNP hydrogel scaffold promoting bone regeneration in osteoporotic bone defects.

In this paper, by organically combining nanoparticles obtained by coupling alendronate sodium with cerium ions, micro-scale hydrogels that offer a suitable environment for cell growth and differentiation, and millimeter-scale tricalcium phosphate capable of providing adequate mechanical properties and mineral salts, a multi-scale biomimetic cerium ion hydrogel scaffold was fabricated. In vitro experiments proved that the 3D-printed tricalcium phosphate-collagen-methacrylate (COMA) hydrogel-Alendronate sodium cerium ion nanoparticles (TCP-H-ACNP) hydrogel scaffold could facilitate osteogenic differentiation of osteoporotic BMSCs and promote angiogenesis. In the *in vivo* model, through establishing a bone defect model in osteoporotic rats and filling the defect area with TCP-H-ACNP hydrogel scaffolds, it exerted an excellent effect on promoting angiogenesis and osteogenesis. Mechanistically, our findings indicate that the TCP-H-ACNP scaffold not only directly promotes osteogenic differentiation of osteoporotic BMSCs via activation of the Wnt signaling pathway but also enhances mitochondrial function in osteoporotic BMSCs by modulating calcium ion transport. This study offers a novel reference for the existing treatment modalities and holds promising prospects for clinical application (Scheme 1).

## 2 Method

### 2.1 Materials

Type I collagen (bovine Achilles tendon source) was obtained from New Material Technology Co., Ltd. (Suzhou Xianjue, China). N-succinimidyl methacrylate ester (NHS-MA) was acquired from Macklin Biochemical Co., Ltd. (Shanghai, China). Pharmaceutical-grade alendronate sodium and cerium (III) chloride heptahydrate (CeCl_3_) were both purchased from Aladdin Industrial Corporation (Shanghai, China).

### 2.2 Preparation of ACNP

Alendronate sodium and Ce ion powder were separately dissolved in ddH_2_O at concentrations of 10 mg/mL (30 μmol/L) and 5 mg/mL (20 μmol/L), respectively. An equal volume of Ce ion powder was slowly dropped into the alendronate sodium solution while vortexing simultaneously. The mixed solution was incubated in a constant-temperature shaker overnight. After the mixture was accomplished, it was centrifuged at 5000 r/min. The precipitate at the bottom of the centrifuge tube was freeze-dried to obtain powder.

Weight yield:

$$\text{Yield}\left(\%\right)=\frac{\text{The}\;\text{actual}\;\text{mass}\;\text{of}\;\text{the}\;\text{obtained}\;\text{nanoparticles}}{\text{Theoretical}\;\text{maximum}\;\text{mass}\;\text{of}\;\text{nanoparticles}}\times 100\%$$

### 2.3 Preparation of collagen-methacrylate (COMA)

**Supplementary material Fig. 1:**
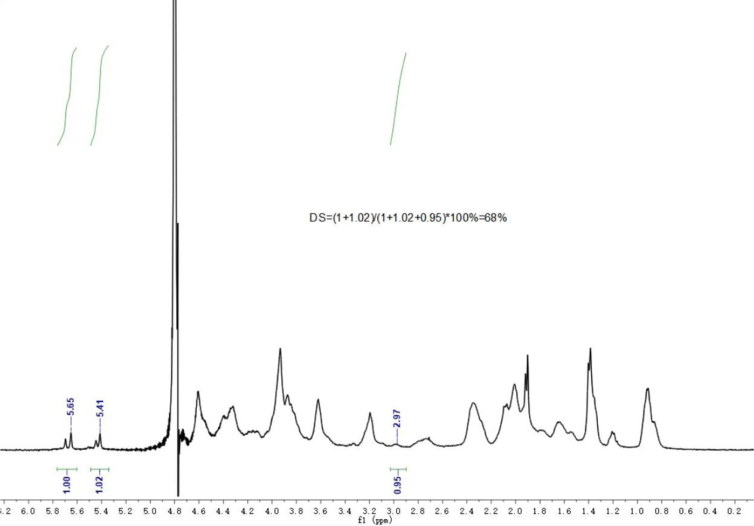
**Characterization of COMA Hydrogel.** Degree of substitution of COMA.

In the preparation of COMA (DS = 68 %) (Supplementary Fig. 1), 5 g of collagen and a stirring bar were added to a round-bottom flask, followed by 100 mL of sterile water to achieve a final collagen concentration of 50 mg/mL. The homogeneous mixture was continuously stirred (500 rpm) at 25 ± 1 °C for 30 min to ensure complete dissolution. The solution pH was carefully titrated to 7.5–8.0 using 2.5 mol/L sodium hydroxide (NaOH) with constant pH monitoring. Separately, 1.000 ± 0.001 g of NHS-MA was precisely weighed in a 15 mL conical tube and dissolved in 2.00 mL anhydrous dimethyl sulfoxide (DMSO) via vortex mixing (2000 rpm, 1 min) followed by 5 min sonication. This NHS-MA/DMSO solution was then introduced to the collagen solution at a controlled rate of 0.5 mL/min using a peristaltic pump while maintaining vigorous stirring (800 rpm). The reaction vessel was immediately wrapped in aluminum foil to protect from light and the conjugation reaction proceeded for 10–12 h at 25 °C with gentle agitation (200 rpm). Post-reaction, the product was purified through dialysis against deionized water (changed every 8 h) for 48 h at 25 °C, followed by freeze-drying for 72 h to obtain the final modified collagen product.

The formula for calculating the degree of substitution:

$$\text{The}\;\text{degree}\;\text{of}\;\text{substitution}=\frac{\text{number}\;\text{of}\;\text{substituted}\;\text{groups}\;}{\text{total}\;\text{number}\;\text{of}\;\text{group}}\times 100\%$$

Number of substituted groups: This refers to the quantity of groups in a molecule that have been substituted through a substitution reaction.

### 2.4 Preparation of TCP-H-ACNP scaffold

The ACNP solution, COMA solution, and photoinitiator were combined to achieve a final concentration of 20 μg/mL for ACNPs and 5 % for the COMA solution. The resulting ACNP-COMA mixture was then carefully poured over the pre-fabricated TCP scaffold to ensure complete impregnation. Cross-linking was subsequently performed under ultraviolet light exposure for a duration of 10 min.

### 2.5 SEM and electron dispersive X-ray (EDX)

After the prepared hydrogel scaffolds and the hydrogel scaffolds after in vitro biomineralization were freeze-dried, they were subjected to SEM scanning for observation. The samples were fixed on the sample stage using conductive adhesive, and a coating was applied using an ion sputtering instrument to enhance conductivity. Then, SEM observation was performed in accordance with the instrument usage specifications. Simultaneously, EDX microanalysis was carried out to determine the composition of the biomineralized particles on the surface of the hydrogel scaffolds.

### 2.6 Rheological properties of hydrogels

Prepare a 50 mg/mL COMA solution using phosphate-buffered saline (PBS). In the COMA-ACNP group, the concentration of ACNP is 20 μg/mL, and add 0.5 mg/mL photoinitiator. After thoroughly mixing the prepared hydrogel solution, take 100–200 μL of the sample onto the sample stage and observe the change curves of the elastic modulus (G′) and viscous modulus (G″) during the gelation process. The sweep frequency range was set to 0.1–100 Hz to simulate the stability of the hydrogel under physiological conditions.

### 2.7 Cell viability

BMSC viability was evaluated using Calcein-AM/PI double staining (Solarbio) after exposure to ACNP particles (5–200 μg/mL) for 1–5 days. Following incubation, cells were stained with 2 μmol/L Calcein-AM and 5 μmol/L PI in PBS (15 min, 37 °C, dark conditions) according to manufacturer instructions. Fluorescence imaging was performed using an inverted fluorescence microscope (Carl Zeiss, Germany) with standard FITC (ex/em: 494/517 nm) and TRITC (ex/em: 535/617 nm) filter sets. Viability was quantified by calculating the percentage of Calcein-AM-positive cells relative to total cells in five random fields per well using ImageJ software. The TCP-H-ACNP treatment groups were evaluated under identical experimental conditions.

### 2.8 Cell proliferation

BMSCs were seeded in standardized density within 96-well culture plates and exposed to gradient concentrations (5–200 μg/mL) of ACNP suspensions. Cellular responses were longitudinally monitored at 24-h intervals over a 7-day experimental timeline (Days 1/3/5/7). Metabolic activity quantification was performed through cell counting kit-8 (CCK-8) colorimetric assay (Dojindo Laboratories, Shanghai) following standardized protocols: Post-treatment cells were incubated with tetrazolium reagent (10 % v/v) for 120 min under physiological conditions (37 °C, 5 % CO_2_). Absorbance values were acquired at 450 nm reference wavelength using a Synergy H1 hybrid multimode reader (BioTek Instruments, Winooski, VT, USA). Comparative evaluation of TCP-H-ACNP composites' proliferative effects was conducted through parallel experimental configurations employing identical methodology.

### 2.9 Animals

Genetically standardized C57BL/6 murine and Sprague Dawley rat cohorts were maintained under SPF conditions within the vivarium facilities of Soochow University Laboratory Animal Center (SULAC). All animal experimentation protocols received formal approval from the Institutional Animal Ethics Committee of Soochow University (Ethics Approval No. SUDA20241225A06), strictly adhering to the National Institutes of Health Guide for the Care and Use of Laboratory Animals (NIH Publication No. 8023), with biosecurity protocols implemented throughout the study.

### 2.10 Construction of ovariectomy (OVX)-induced mouse/rat osteoporosis model

Experimental animals were anesthetized with isoflurane gas (RWD, China, Cat. No. R510-22-10). Following the induction of anesthesia, the subjects were secured in a supine posture on the surgical platform, and the dorsal fur was removed. A midline dorsal incision, approximately 1 cm in length, was created using sterile surgical scissors. Subsequently, the subcutaneous and muscular layers were gently separated to reveal the underlying musculature.

An additional incision, positioned 1 cm caudal to the spinal rib margin, was made through the lumbar muscle, exposing the periovarian adipose tissue adjacent to the uterine horn. The adipose compartment was carefully extracted, revealing the ovarian structure characterized by its distinct cauliflower-like morphology.

Ligation was then performed at the proximal and distal regions of the uterine horn near the oviduct. The periovarian adipose tissue was precisely dissected, followed by transection of the uterine horn and complete ovarian excision. Finally, the adipose tissue was repositioned into the abdominal cavity, and the peritoneal and muscular layers were sutured sequentially. The skin was closed to conclude the surgical procedure.

### 2.11 Construction of distal femur defect model in OVX-induced rat osteoporosis

Firstly, an osteoporosis model of rats was constructed. After two months, the successfully constructed OVX-induced rat osteoporosis were randomly divided into four groups: the defect group, the defect + TCP group, the defect + TCP-H group, and the defect + TCP-H-ACNP group. A cortical defect of approximately 3∗3∗1 mm (length ∗ width ∗ depth) was ground on the right femur of the rats in the bone defect model group using a high-speed cranial drill. The defect sites were filled with hydrogel scaffolds, and the wounds were sutured layer by layer. The day of model establishment was defined as day 0. Tissue samples were collected at 4 weeks and 8 weeks for subsequent experiments.

### 2.12 Preparation of primary mouse/rat derived osteoporosis BMSCs

The study employed OVX rodent models (C57BL/6 mice and Sprague–Dawley rats) for osteoporosis induction. After 8-week post-operative confirmation of osteoporotic phenotypes via micro-CT analysis, BMSCs were aseptically isolated from bilateral femoral medullary cavities. Primary cultures were established using α-minimum essential medium (α-MEM; Hyclone, Logan, UT) supplemented with 10 % fetal bovine serum (FBS; Gibco, Australia origin) and 1 % penicillin-streptomycin (Gibco), with media changes every 48 h. Cells were maintained at 37 °C in a humidified 5 % CO_2_ incubator (Thermo Scientific) until 80–90 % confluence. Nutrient replenishment was performed at 48-h intervals through complete medium replacement. For subsequent experimental applications, first-passage BMSCs from OVX mice and second-passage BMSCs from OVX rats were selectively employed in accordance with experimental design requirements.

### 2.13 ALP and alizarin red staining and quantification

Following initial co-culture with biomaterial elution medium under hypoxia-mimetic conditions (5 % CO_2_, 37 °C), BMSCs were maintained in osteoinductive medium with scheduled medium replacement cycles maintained at 72-h intervals. At the primary mineralization phase (Day 7), alkaline phosphatase (ALP) enzymatic activity was assessed through dual-modality analysis: Histochemical detection using 5-bromo-4-chloro-3-indolyl phosphate ester p-toluidine salt/Tetranitroblue tetrazolium chloride (BCIP/NBT) substrate chromogenic system (Beyotime Biotechnology, Shanghai, China) complemented with biochemical quantification via p-nitrophenyl phosphate (pNPP) hydrolysis assay (Jiancheng Bioengineering Institute, Nanjing, China). Both analytical procedures were executed following manufacturers' optical density calibration protocols, with enzymatic activity values normalized to total cellular protein content. Following 21 days of osteogenic induction, calcium deposition was quantified through Alizarin Red S (ARS) staining (Solarbio, China). The ARS solution (1 % w/v, pH 4.2) specifically chelates with calcium phosphate deposits, forming characteristic orange-red complexes. Cells were first fixed with freshly prepared 4 % paraformaldehyde (Biosharp, China) in PBS for 10 min at room temperature (25 ± 1 °C), then incubated with ARS solution for 20 min with gentle agitation (50 rpm). After thorough washing with ddH_2_O to remove unbound dye, mineralized nodules were documented using bright-field microscopy (Olympus IX83, Japan). For quantitative analysis, the stained calcium deposits were dissolved in 10 % cetylpyridinium chloride (CPC) for 30 min, and absorbance was measured at 562 nm using a microplate reader (BioTek, USA).

### 2.14 Quantitative real-time polymerase chain reaction (RT-qPCR)

RNA isolation was performed utilizing dual methodologies: column-based purification (Cell & Tissue RNA Extraction Kit, Vazyme Biotech Co., China) and organic solvent extraction (Trizol reagent). Subsequently, reverse transcription processes were conducted with the Hisscript Ⅲ qPCR-Optimized RT SuperMix system (Vazyme Biotech Co., China) to generate complementary DNA templates. Quantitative gene expression profiling was executed via SYBR Green chemistry using Premix ExTaqTM master mix (Takara Bio, China) on a real-time PCR platform. Amplification data were computationally processed through the comparative threshold cycle (2^−^^ΔΔCT^) algorithm with *glyceraldehyde-3-phosphate dehydrogenase (GAPDH)* serving as endogenous normalization control. Target-specific oligonucleotide primers for *collagen type I alpha 1 chain (Col1a1)*, *runt-related transcription factor 2 (RUNX2)*, and metabolic regulators (*Adcyap1r1, Atp2a3*, etc.), along with reference gene primers, are comprehensively cataloged in Supplementary Table 1.

### 2.15 Western Blot

BMSCs isolated from OVX-mice/rat were seeded in 6-well plates at a density of 1–2 × 10^5^ cells per well. Upon reaching 80–90 % confluency, the culture medium was supplemented with material leachate. Protein extraction was performed at predetermined time intervals.

Protein concentrations were quantified using a BCA assay, followed by separation on a 10 % SDS-polyacrylamide gel. Electrophoresis was conducted to resolve target protein bands, which were subsequently probed with primary antibodies overnight. Following incubation with HRP-conjugated secondary antibodies, protein bands were detected by chemiluminescence using Superpico ECL Master Mix (Vazyme, China). Quantitative analysis of band intensity was performed through densitometry with ImageJ software.

### 2.16 Micro-computed tomography scanning

Post-interventional specimen collection was chronologically stratified: Murine femoral specimens were harvested at 1-month post-systemic drug administration, while rat osteotomy models underwent bilateral femur retrieval at dual endpoints (4- and 8-week post-defect induction). All specimens underwent high-resolution micro-CT scanning (SkyScan 1272, Bruker MicroCT, Belgium) with species-specific parameterization: Murine scans achieved 9 μm isotropic voxel resolution, whereas rat specimens were imaged at 18 μm spatial resolution, maintaining standardized acquisition parameters (50 kv X-ray potential, 200 μA beam current, 0.7° rotational increment through 180° angular range).Volumetric reconstruction was conducted through NRecon v1.7.4.2 software (Bruker), followed by 3D morphological rendering via Mimics Innovation Suite v10.01 (Materialise NV, Leuven). Quantitative morphometry focused on subchondral plate parameters: Bone mineral density (BMD, mg cm^−3^) and bone volume per bone as a percentage of total tissue volume (BV/TV, %) of the subchondral bone plate were analyzed.

### 2.17 ROS staining

The osteoporotic rat-derived BMSCs were treated with DCFH-DA (10 μM) in serum-free DMEM/α medium at 37 °C in the dark for 30 min using an active oxygen (ROS) detection kit (Beyotime, China, S0033 M). Images were captured using an inverted fluorescence microscope (Zeiss, Axio Observer Z1, Germany).

### 2.18 Determination of cerium ion release

The TCP-H-ACNP hydrogel scaffolds were incubated in PBS solution within centrifuge tubes, which were then transferred to a 37 °C constant-temperature shaker. At designated time points, the supernatant was carefully extracted from the tubes, and the cerium ion concentration was quantified using inductively coupled plasma optical emission spectrometry/mass spectrometry (ICP-OES/MS; Thermo Fisher).

### 2.19 Statistical analysis

Quantitative datasets were processed through parametric descriptive analyses, with all experimental measurements expressed as arithmetic mean ± standard deviation (SD). Statistical evaluations were performed utilizing GraphPad Prism 8.0 statistical suite (GraphPad Inc., San Diego, CA), implementing hypothesis-testing protocols as follows: For comparative analysis between two experimental cohorts, a two-tailed unpaired Student's t-test was systematically applied. Multivariate comparisons across intervention groups employed single-factor analysis of variance (ANOVA) with post hoc verification. Experimental outcomes were considered statistically significant when achieving a probability threshold of P < 0.05, with asterisk notation (∗) denoting significance levels in graphical representations.

## 3 Results

### 3.1 Characterization and biocompatibility assessment of nanoparticle ions

**Fig. 1:**
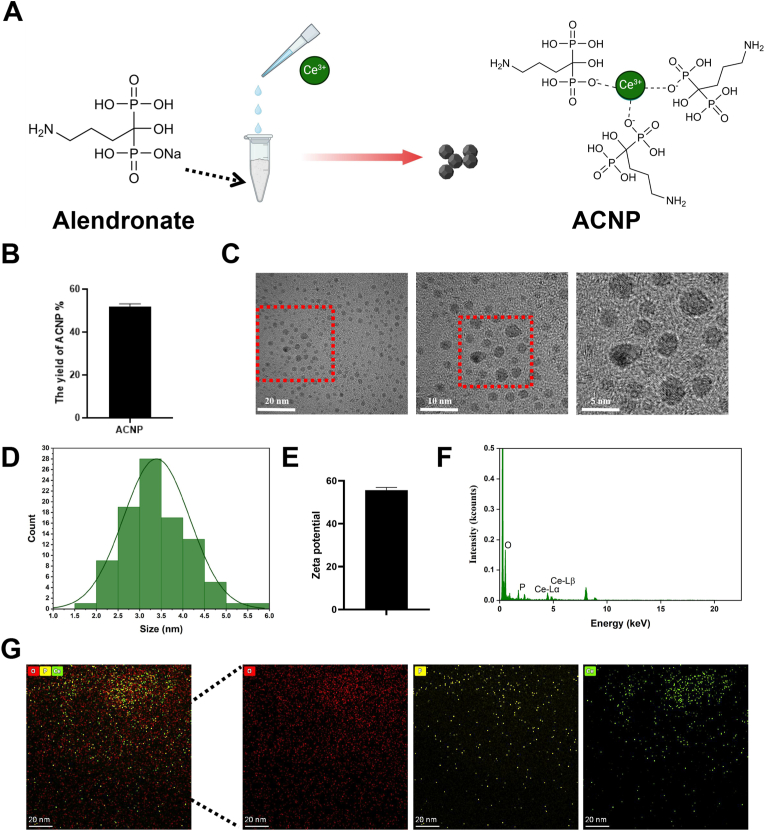
**Morphology and characterizations of ACNP. (A)** A schematic diagram of material preparation. **(B)** Weight yield of ACNP. **(C)** TEM image of ACNP. **(D)** Size distribution of ACNP. **(E)** Zeta potential of ACNP. **(F**–**G)** TEM-EDS elemental analysis of ACNP.

**Supplementary material Fig. 2:**
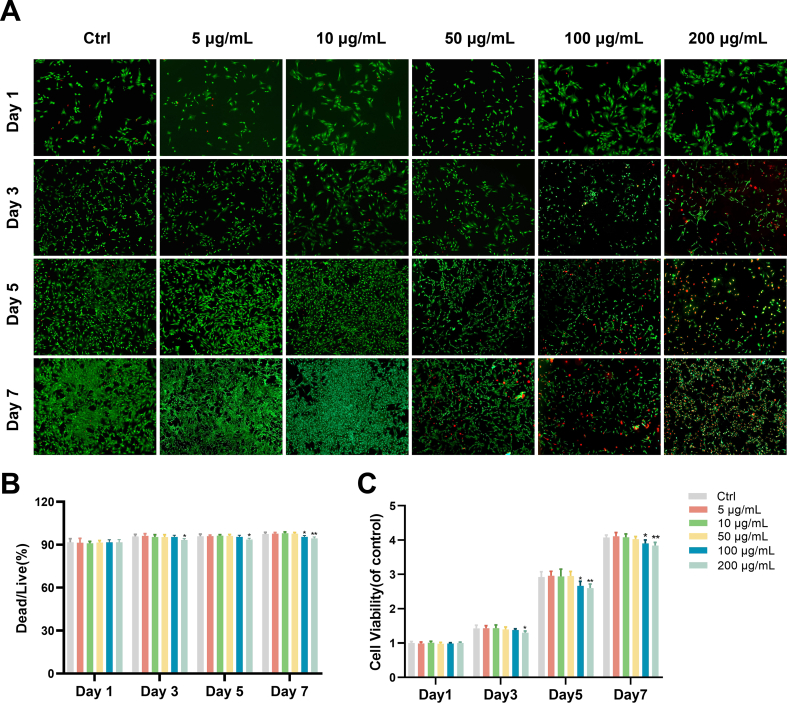
**The biocompatibility of different concentrations of ACNP.(A)** The cell viability of BMSCs cultured cultured by different concentrations of ACNP (0 μg/mL, 5 μg/mL, 10 μg/mL, 50 μg/mL, 100 μg/mL, 200 μg/mL) were determined using the Live/Dead Cell staining, Scale bar = 1 mm. **(B)** Quantification of the live cells cultured by different concentrations of ACNP, n = 3. **(C)** Cell proliferation rate was determined by CCK-8 assays, n = 4.

Through vortex oscillation, we obtained the nanoparticles of alendronate sodium-cerium ion (Fig. 1A). The actual yield of nanoparticles obtained through this method is approximately 51 % (Fig. 1B). Through TEM, the ultrastructure of the nanoparticles was observed (Fig. 1C). The particle size of ACNP particles displayed in the TEM images was quantitatively shown by Image J. The size of ACNP particles was mainly concentrated in the range of 2.5–4.5 nm (Fig. 1D). The zeta potential mean value of ACNP is 57 mV. Compared with alendronate sodium and CeCl_3_, it shows better stability (Fig. 1E). Through elemental analysis, cerium ion was combined with alendronate (Fig. 1F–G). *In* *vitro* safety test of ACNP particles, we screened the appropriate concentration of ACNP through live/dead staining. The results showed that when the concentration of ACNP was greater than 50 ug/mL, it exhibited obvious toxic effects on BMSCs (Supplementary Fig. 2A–B). Through the CCK-8 assay, we found that when the concentration of ACNP particles was greater than 100 μg/mL, it had a significant inhibitory effect on cell proliferation (Supplementary Fig. 2C). These results guided us to search for an appropriate concentration within the range of 10–50 μg/mL. Finally, 20 μg/mL was determined as the final working concentration.

### 3.2 Nanoparticles enhance the *invitro* osteogenic differentiation of osteoporotic mouse-derived BMSCs

**Fig. 2:**
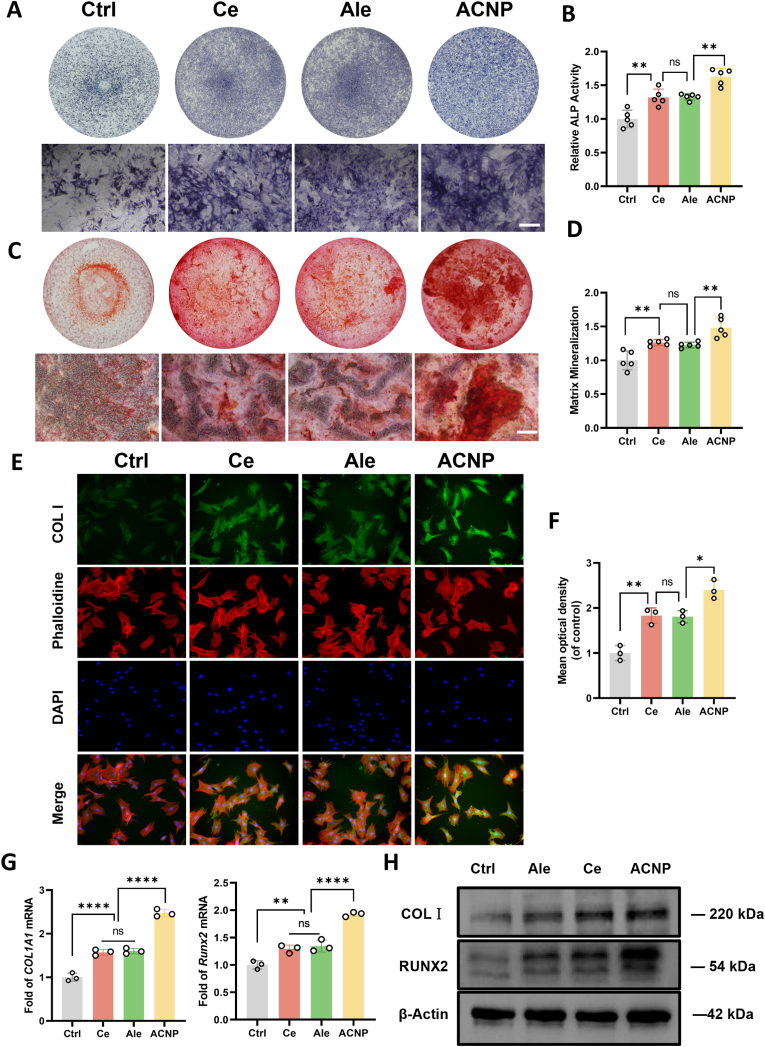
**Nanotherapeutic Enhancement of Osteogenic Potential in Osteoporotic BMSCs. (A)** Histochemical detection of ALP activity in OVX-induced osteoporotic BMSCs following 7-day osteoinductive culture (n = 5 biological replicates; Scale bar: 100 μm). **(B)** Spectrophotometric quantification of ALP enzymatic activity normalized to total cellular protein (n = 5). **(C)** ARS staining after 21-day differentiation protocol (n = 5 independent experiments; Scale bar: 100 μm). **(D)** Quantitative mineralization analysis through ARS destaining spectrophotometry (n = 5). **(E)** COL I cytoskeletal architecture visualization by immunofluorescence confocal microscopy (Scale bar: 50 μm). **(F)** Semi-quantitative analysis of COL I fluorescence intensity using ImageJ software (n = 3). (G) Transcriptional regulation of osteogenic markers (*Runx2*, *Col1a1*) quantified through quantitative reverse transcription PCR (n = 3). **(H)** Western immunoblotting validation of COL I and RUNX2 protein expression levels (n = 3 biological replicates). Statistical significance thresholds: ∗*p* < 0.05, ∗∗*p* < 0.01 (two-tailed ANOVA with Tukey post hoc).

To investigate whether ACNP particles can more directly promote bone formation in an osteoporotic environment compared to individual Ce ions and alendronate, we conducted in vitro treatment of osteoporotic mouse-derived BMSCs with ACNP particles, Ce ions, and alendronate solution. ALP staining demonstrated the osteogenic potential of ACNP particles, showing enhanced differentiation of osteoporotic mouse-derived BMSCs (Fig. 2A). Quantitative analysis revealed a 20 % higher ALP activity in the ACNP-treated group compared to both Ce ions and alendronate (Fig. 2B). Furthermore, ARS staining after 21-day induction showed increased mineralization nodule formation in ACNP-treated cultures (Fig. 2C). Densitometric measurements indicated superior calcium deposition in the ACNP group, with 16.7 % and 20.0 % greater levels than Ce ions and alendronate, respectively (Fig. 2D). Quantitative analysis revealed that both Ce ions and alendronate sodium significantly increased the expression of COL I protein, with respective increments of 75.0 % and 70.0 % compared to the control group. Notably, ACNP particles exhibited an enhanced ability to do so, resulting in a 130 % increase (Fig. 2E and F). Moreover, the bone formation markers in the ACNP-co-cultured osteoporotic mouse-derived BMSCs exhibited a significant increase. In comparison with the control group, there was a 150 % and 140 % respective increase in the transcription levels of *Col1a1* and *Runx2* (Fig. 2G). The Western Bolt results demonstrated that Ce ion, alendronate sodium, and ACNP particles were effective in addressing the deficiency of COL I and RUNX2 proteins in osteoporotic mouse-derived BMSCs, with ACNP particles exhibiting the most pronounced effect (Fig. 2H). These findings indicate that ACNP particles have the potential to restore the reduced osteogenic differentiation capability of osteoporotic mouse-derived BMSCs, and this capability is further significantly enhanced by the presence of Ce ions and alendronate alone.

### 3.3 Nanoparticles facilitate angiogenesis in human umbilical vein endothelial cells (HUVECs) cultured *invitro*

**Fig. 3:**
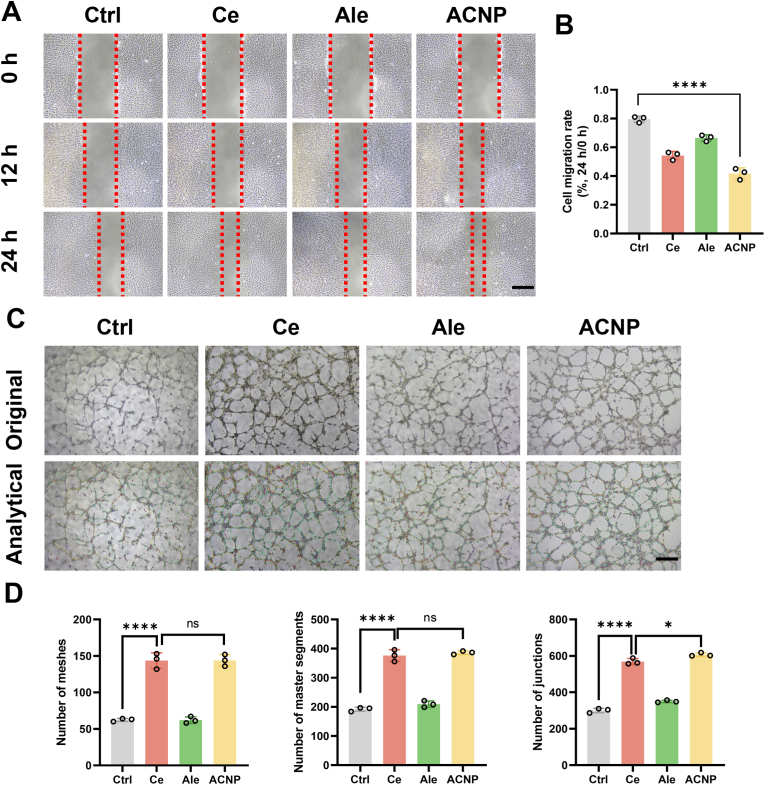
**Nanoparticles facilitate angiogenesis in HUVECs cultured *in vitro*. (A**–**B)** Representative micrographs and corresponding quantitative analysis of the wound closure assay (Scale bar: 1 mm). **(C)** Microscopic images depicting endothelial tube formation (Scale bar: 100 μm). **(D)** Morphometric evaluation of tubular networks, including total mesh area, segment length, and branch point density (n = 3 biological replicates). Statistical significance thresholds: ∗*p* < 0.05, ∗∗*p* < 0.01, ∗∗∗*p* < 0.001, ∗∗∗∗*p* < 0.0001 (two-tailed ANOVA with Tukey post hoc).

**Supplementary material Fig. 3:**
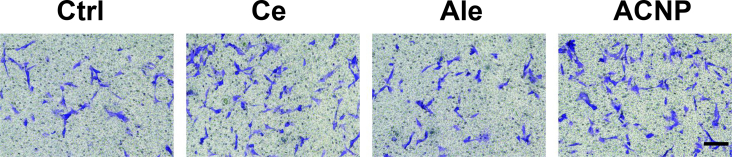
**ACNP enhances the migration of BMSCs.** The representative images and quantification of HUVECs migration. Scale bar = 100 μm.

The development of local microvessels at the fracture site is crucial for the prognosis of osteoporotic fractures. Therefore, in addition to preserving the osteoporotic mouse-derived BMSCs' osteogenic differentiation capability, we also focus on their angiogenic differentiation ability to ensure enhanced osteogenesis. We treated HUVECs with Ce ions, alendronate sodium, and ACNP particles separately, leading to the differentiation of the three groups of HUVECs into angiogenic cells. The results of the cell scratch assay demonstrated that HUVECs treated with ACNP particles exhibited enhanced promotion of migration at 8 and 24 h post-treatment (Fig. 3A). 24 h Quantitative analysis revealed that, compared to the control group, the Ce ion group, alendronate sodium group, and ACNP particle group showed increases of 31.3 %, 18.8 %, and 50 %, respectively (Fig. 3B). The findings indicated that alendronate sodium did not exert a significant impact on HUVEC angiogenesis, whereas cerium ions and ACNP particles both facilitated the *in* *vitro* angiogenic differentiation of HUVECs (Fig. 3C). Quantitative analysis revealed significant increases in mesh, master segments, and junctions following treatment. Specifically, the cerium ion group exhibited enhancements of 1.54-fold, 95 %, and 93 % relative to the control. Similarly, the ACNP particle group demonstrated comparable improvements, with increases of 1.54-fold, 97 %, and a doubling (1 × ) in the respective measurements (Fig. 3D). Crystal violet staining analysis further demonstrated that HUVEC migration was significantly promoted in both the Ce particle and ACNP particle treatment groups relative to the control. However, no statistically significant disparity was detected between the two experimental groups. However, the Alendronate group did not demonstrate a capacity to promote HUVECs migration. The above results suggest that the promotion of angiogenesis and vascular migration *in* *vitro* by ACNP particles is primarily attributed to cerium ions, with alendronate demonstrating minimal impact in this aspect (Supplementary Fig. 3A).

### 3.4 Intraperitoneal injection of ACNP nanoparticles improves bone loss in osteoporotic mice

**Fig. 4:**
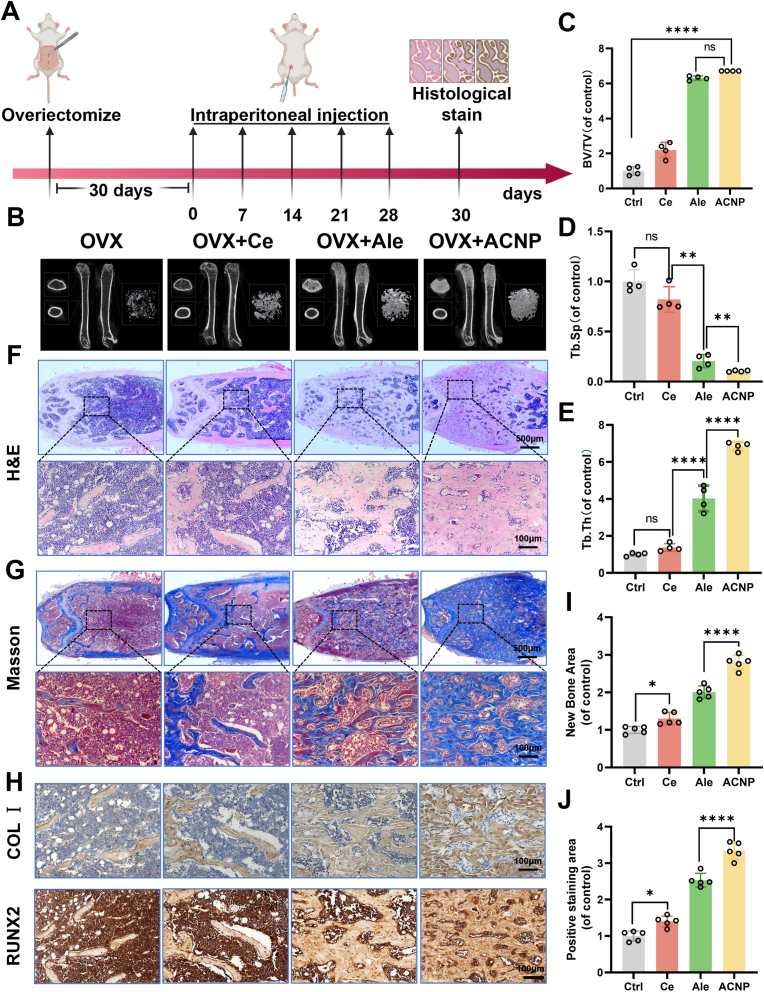
**Evaluation of bone regeneration efficacy in osteoporotic femoral models following 4-week therapeutic intervention. (A)** Schematic representation of experimental model establishment and tissue collection protocol. **(B)** Three-dimensional micro-CT reconstruction analyses were conducted to evaluate neovascularization in the distal femur region (n = 4). **(C**–**E)** Histomorphometric quantification parameters including bone volume fraction (BV/TV), intertrabecular distance (Tb.Sp), and trabecular structural thickness (Tb.Th) were systematically measured in distal femoral specimens (n = 4). **(F)** Histopathological examination using H&E staining visualized tissue morphology (n = 6). **(G&I)** Modified trichrome staining (MTS) demonstrated collagen deposition patterns in bone repair areas (n = 5). **(H**–**J)** Immunohistochemical detection of COL I and RUNX2 expression (Scale bar: 100 μm). Statistical significance thresholds: **∗***p* < 0.05, ∗∗*p* < 0.01 (two-tailed ANOVA with Tukey post hoc).

Subsequently, we verified the *in vivo* osteogenic performance of ACNP particles in the mouse osteoporosis model induced by OVX surgery. ACNP particles were intraperitoneally injected once a week, and femoral specimens were collected after one month (Fig. 4A). Ce ions, alendronate sodium, and the ACNP particle solution were intraperitoneally injected once a week. Femoral specimens were collected one month later. Micro-CT evaluation of the distal femur in OVX-induced osteoporotic mice revealed substantial bone loss compared to controls (Fig. 4B). Notably, therapeutic intervention with Ce ions, alendronate sodium, or ACNP particle solutions effectively attenuated this OVX-induced bone deterioration. Quantitative assessments demonstrated significant improvements in key parameters relative to the untreated OVX group: both bone volume fraction (BV/TV) and trabecular thickness (Tb.Th) exhibited marked increases, whereas trabecular spacing (Tb.Sp) showed a pronounced reduction across all treatment groups (Fig. 4C–E). Among them, the improvement in the ACNP particle injection group was more pronounced. Histological staining further confirmed the *in vivo* osteogenic role of ACNP particles. Through Hematoxylin and Eosin (H&E) staining, it was discovered that ACNP particle injection could significantly ameliorate the trabecular bone reduction in OVX mice, and the effect of nanoparticles was significantly stronger than that of individual Ce ions and alendronate sodium injection (Fig. 4F). The results of Masson staining indicated that intraperitoneal injection of ACNP particles could enhance the deposition of collagen, and the volume of newly generated bone was significantly increased (Fig. 4G and I). Immunohistochemical staining for type I collagen simultaneously revealed that the ACNP group had better collagen formation (Fig. 4H and J). The above results indicate that intraperitoneal injection of Ce ions, alendronate sodium, and ACNP particle solution can significantly improve the bone loss caused by OVX-induced osteoporosis mice, among which the improvement effect of ACNP particle group is the most obvious. *In vivo*, it validates the osteogenic effect of ACNP particles.

### 3.5 Preparation and characterization identification of multi-scale release fusion TCP-H-ACNP hydrogel scaffolds

**Fig. 5:**
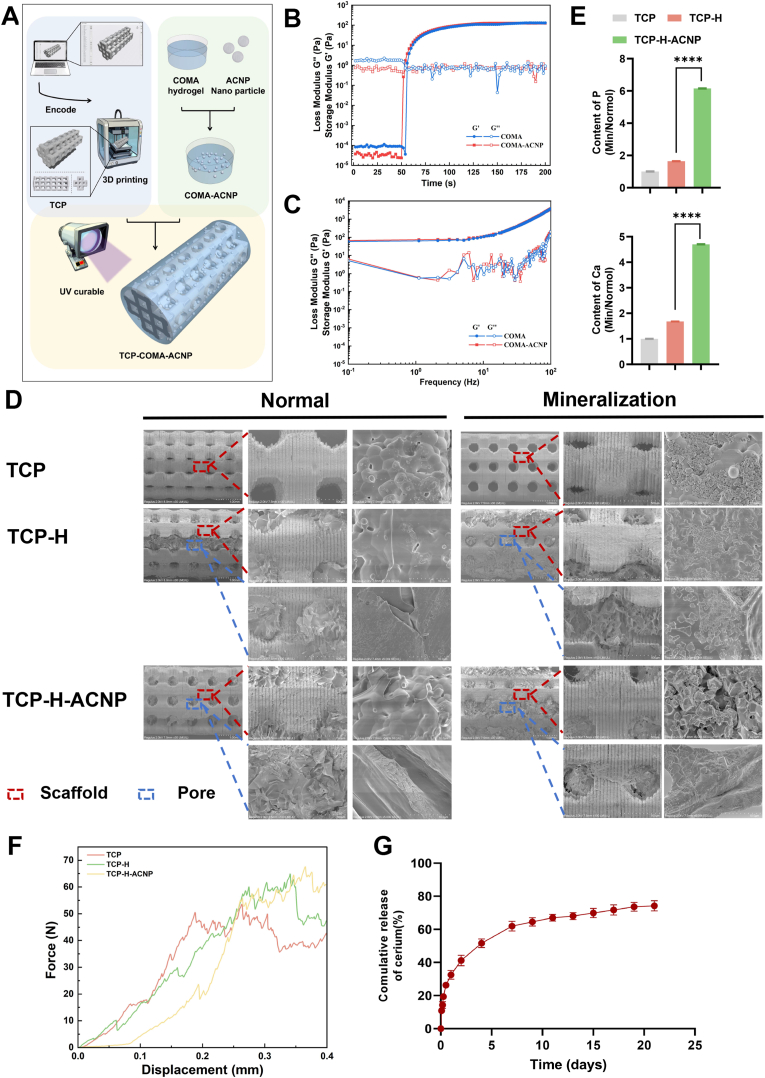
**Morphology and characterizations of the scaffold TCP-H-ACNP. (A)** The fabrication process of TCP-H-ACNP hydrogel scaffolds. **(B)** The evolution of storage modulus (G′) and loss modulus (G″) of COMA and COMA-ACNP hydrogels with gelation time at 1.59 Hz and 1 % strain (37 °C, PBS). **(C)** The evolution of storage modulus (G′) and loss modulus (G″) of COMA and COMA-ACNP hydrogels with sweep frequency (0.1–100 Hz, 1 % strain) under physiological conditions (37 °C, PBS environment). **(D)** SEM image of the TCP-H-ACNP scaffold and Bio-mineralized ACNP scaffold. **(E)** The variations of calcium and phosphorus before and after biomineralization. **(F)** The mechanical compression performance of TCP, TCP-H and TCP-H-ACNP scaffold. **(G)** Cumulative release curve of cerium ions. Statistical significance thresholds: ∗*p* < 0.05 or ∗∗∗∗*p* < 0.0001 (two-tailed ANOVA with Tukey post hoc).

**Supplementary material Fig. 4:**
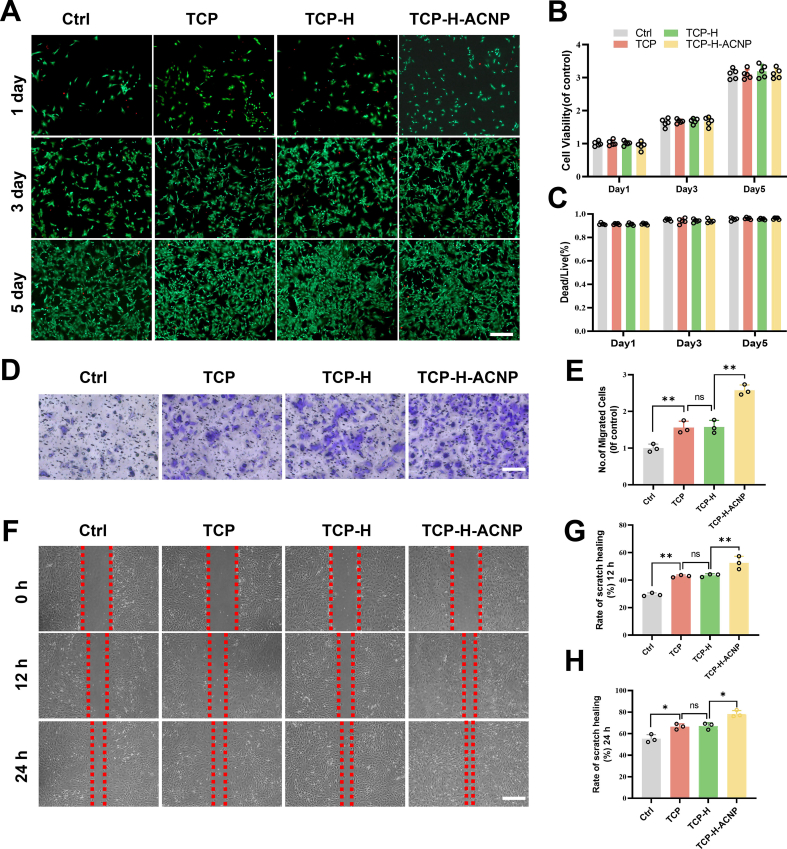
**The biocompatibility ofTCP-H-ACNP. (A)** The cell viability of BMSCs cultured cultured on TCP, TCP-H, and TCP-H-ACNP were determined using the Live/Dead Cell staining, Scale bar = 1 mm. **(B)** The proportion of live cells to dead cells. **(C)** Cell proliferation rate was determined by CCK-8 assays, n = 4. **(D**–**E)** The representative images and quantification of BMSCs migration. Scale bar = 100 μm. **(F**–**H)** The representative images and quantification of wound healing experiment. Scale bar = 1 mm. Statistically significant differences were indicated by ∗ *p* < 0.05 or ∗∗*p* < 0.01.

Through *in vivo* and *vitro* experiments, we have verified the osteogenic and angiogenic functions of ACNP particles. However, in contrast to the *in vivo* model of osteoporosis, the *in vivo* model of bone defects confronts more complex local circumstances. Sufficient mechanical support and a suitable local microenvironment for cell growth significantly influence the repair of bone defect sites. Thus, pure ACNP particles fail to meet these requirements satisfactorily. Therefore, we designed a TCP scaffold with a porous structure and realized it through 3D printing technology. We prepared COMA hydrogel using type I collagen from bovine Achilles tendons. We combined the two with the ACNP particles prepared as mentioned above. A multi-scale biomimetic scaffold with millimeter-micron-nanometer dimensions was constructed. The millimeter-level TCP scaffold can offer sufficient mechanical support for the defect site. The micron-level COMA hydrogel can provide a suitable microenvironment for cell growth at the defect site. And the nanoparticles promote the local osteogenic capacity by exerting their biological effects (Fig. 5A). After the hydrogels scaffolds were prepared, we further tested their performance. The addition of ACNP particles did not change the gelation time and strength of the COMA hydrogel (Fig. 5B). Under physiological conditions (37 °C, PBS environment), we found that both COMA and COMA-ACNP hydrogels exhibited good elasticity properties. There was no significant difference between the pure COMA hydrogel group and the COMA-ACNP group (Fig. 5C). It can be observed via SEM that the pores of the TCP-H and TCP-H-ACNP scaffolds are filled with hydrogel. Meanwhile, the SEM results of in vitro mineralization demonstrate that TCP-H-ACNP has a stronger mineralization ability. The EDS scan of the mineral deposits on the surface reveals that, compared with the TCP group, the deposition ratios of calcium ions and phosphate ions in the TCP-H-ACNP group increase by 3.5 times and 5 times, respectively (Fig. 5D–E). After co-culturing the scaffold with BMSCs, tests for cell viability and proliferation were conducted. The results of live/dead staining indicated that the scaffold materials were non-toxic to BMSCs (Supplementary Fig. 4A and C). It was found through the CCK-8 assay that there was no obvious effect on proliferation (Supplementary Fig. 4B). The results of crystal violet staining indicated that compared with the control group, the TCP, TCP-H, and TCP-H-ACNP groups were all conducive to the migration of BMSCs (Supplementary Fig. 4D–E). The mechanical compression results show that the TCP scaffold, due to its trabecular-like structure, exhibits a segmented fracture pattern on the curve. TCP-H and TCP-H-ACNP, with the filling of COMA hydrogel, demonstrate a better compressive load-bearing capacity at the final fracture compared to the TCP group, approximately (61.33 ± 2.86) N. Based on the surface area of the TCP stent, we calculated its pressure to be approximately (8.683 ± 0.413) MPa (Fig. 5F). The biological performance of TCP-H-ACNP scaffolds depends on the degradation of COMA hydrogel to produce ACNP. We determined the cumulative release of cerium ions through ICP experiments. The results showed that the release rate of cerium ions reached a stable state at 2 weeks (Fig. 5G). The results of the scratch assay at 12 h and 24 h also revealed the same trend, with increases of 45 %, 48 %, and 78 % at 12 h and 20 %, 21 %, and 42 % at 24 h, respectively (Supplementary Fig. 4F and H).

### 3.6 The scaffold promoted the *invitro* osteogenic differentiation of osteoporotic rat-derived BMSCs

**Fig. 6:**
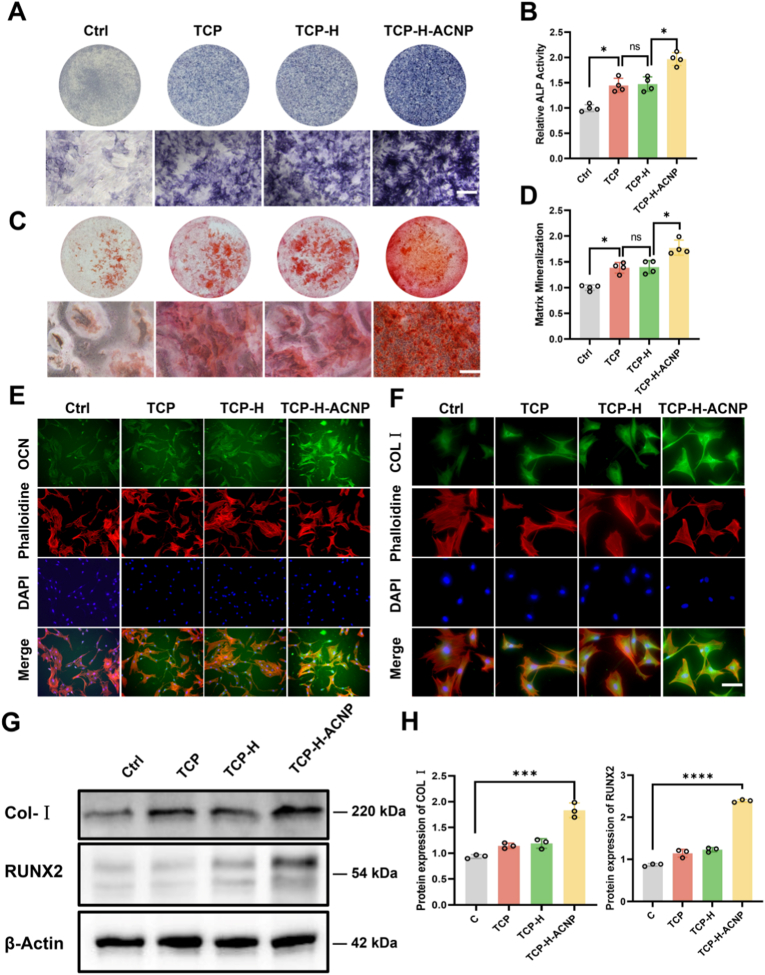
**The scaffold promoted the *invitro* osteogenic differentiation of osteoporotic rat-derived BMSCs. (A)** Histochemical detection of ALP activity in osteoporotic rat-derived BMSCs following 7-day osteoinductive culture (n = 5 biological replicates; Scale: 100 μm). **(B)** Spectrophotometric quantification of ALP enzymatic activity normalized to total cellular protein (n = 4). **(C)** ARS staining after 21-day differentiation protocol (n = 6 independent experiments; Scale: 100 μm). **(D)** Quantitative mineralization analysis through ARS destaining spectrophotometry (n = 4). **(E&F)** OCN and COL I cytoskeletal architecture visualization by immunofluorescence confocal microscopy (Scale: 50 μm). **(G)** Western immunoblotting validation of COL I and RUNX2 protein expression levels (n = 3 biological replicates). **(H)** Quantitative Assessment of COL I and RUNX2 Protein Expression Levels. Statistical significance thresholds: ∗*p* < 0.05, ∗∗*p* < 0.01 (two-tailed ANOVA with Tukey post hoc).

**Supplementary material Fig. 5:**
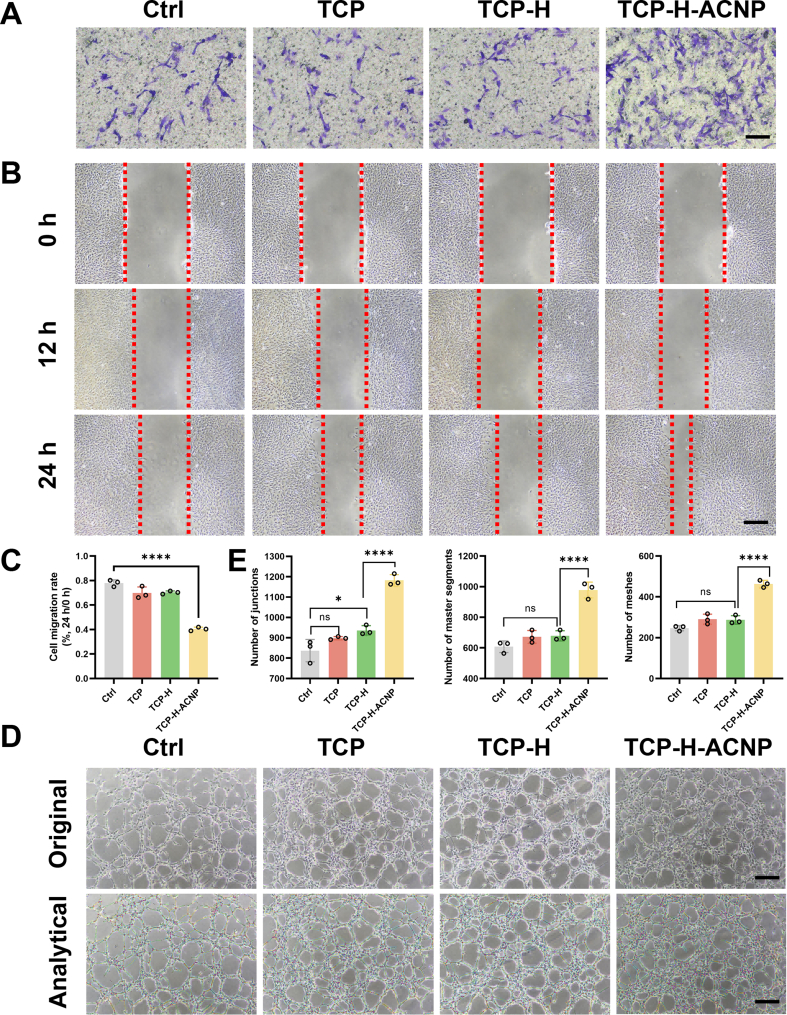
**The scaffolds facilitate angiogenesis in HUVECs cultured in *vitro*. (A)** The representative images and quantification of HUVECs migration. Scale bar = 100 μm. **(B**–**C)** The representative images and quantification of wound healing experiment. Scale bar = 1 mm. **(D)** The representative images of tube formation. Scale bar = 100 μm. **(E)** Quantification of the meshes, master segments and junctions, n = 4. Statistically significant differences were indicated by ∗ *p* < 0.05, ∗∗*p* < 0.01, ∗∗∗*p* < 0.001, ∗∗∗∗*p* < 0.0001.

Upon the completion of the construction of the multi-scale biomimetic hydrogel scaffold, in order to test its biological performance, the leachates of TCP, TCP-H, and TCP-H-ACNP scaffolds were co-cultured with osteoporotic rat-derived BMSCs for osteogenic differentiation, respectively. The results of ALP staining indicated that, compared with the control group, all the TCP, TCP-H, and TCP-H-ACNP scaffold groups could significantly promote the secretion of alkaline phosphatase. After quantitative analysis, it was shown that the activities of ALP increased by 45 %, 48 %, and 97 %, respectively (Fig. 6A and B). After 21 days of osteogenic differentiation, the results of alizarin red staining and quantitative analysis demonstrated that all the three scaffold groups could significantly promote the formation of mineralized nodules, increasing by 39 %, 40 %, and 78 %, respectively (Fig. 6C and D). Simultaneously, the results of cellular immunofluorescence staining of osteogenesis-related proteins revealed that the TCP-H-ACNP hydrogel scaffold could significantly increase the expressions of osteogenesis-related proteins OCN and COL 1 (Fig. 6E and F). The results of Western blot also indicated that TCP-H-ACNP could significantly increase the expressions of COL 1 and RUNX2 (Fig. 6G and H). Meanwhile, in terms of angiogenesis, the composite hydrogel also inherits the angiogenic-promoting ability of ACNP (Supplementary Fig. 5A–E). The above results suggest that the multi-scale biomimetic hydrogel scaffold has a distinct role in promoting osteogenic differentiation and angiogenesis *in* *vitro*.

### 3.7 The TCP-H-ACNP scaffold influences osteogenic differentiation through remodeling the extracellular matrix components of osteoporotic rat-derived BMSCs and affecting calcium ion transport

**Fig. 7:**
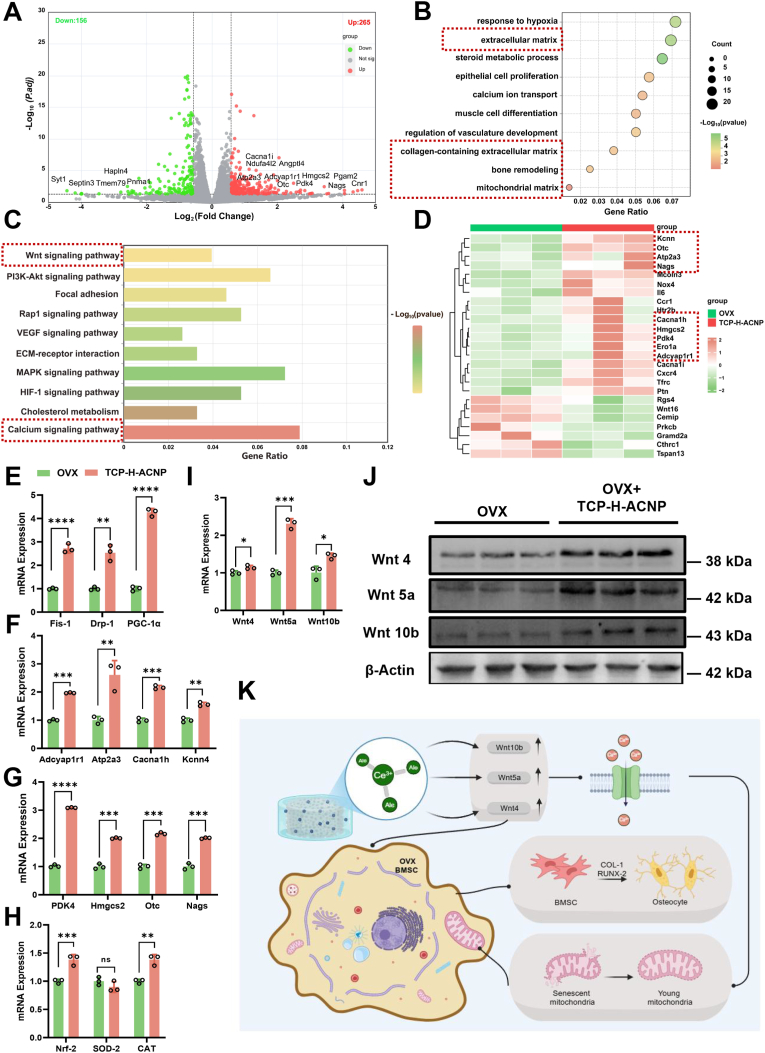
**The TCP-H-ACNP scaffold influences osteogenic differentiation through remodeling the extracellular matrix components of osteoporotic rat-derived BMSCs and affecting calcium ion transport. (A)** Volcano plots display the differentially expressed genes. **(B)** Bubble plots reflecting the outcomes of GO analysis. **(C)** Enrichment analysis of KEGG signaling pathways. **(D)** Heatmap of extracellular matrix composition and calcium ion transport differentially expressed genes. **(E)** The expression of Mitochondrial function-related genes *Fis-1, Drp-1*, and *Pgc-1α*. **(F)** The expression of calcium ion transport-related genes *Adcyap1r1, Atp2a3, Cacna1h* and *Kcnn4*, n = 3. **(G)** The expression of extracellular matrix composition-related genes *Pdk4, Hmgcs2, Otc, and Nags*, n = 3. **(H)** The expression of antioxidant-related genes *Nrf-2, Sod-2,* and *Cat.***(I)** The expression of Wnt Signal pathway-related genes. **(J)** The expression of Wnt Signal pathway-related protein. **(K)** The TCP-H-ACNP hydrogel scaffold promotes osteogenic differentiation of OVX-BMSCs via the Wnt signaling pathway. Meanwhile, it can enhance the mitochondrial function of OVX-BMSCs by facilitating the transport of Ca particles, thereby boosting the osteogenic differentiation potential of OVX-BMSCs. Statistical significance thresholds: ∗*p* < 0.05 or ∗∗*p* < 0.01 (two-tailed ANOVA with Tukey post hoc).

**Supplementary material Fig. 6:**
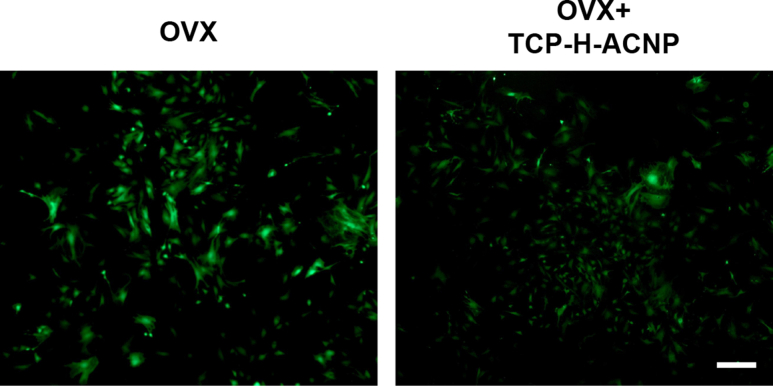
**The TCP-H-ACNP scaffold can reduce the production of ROS in OVX-BMSCs.** The representative images of ROS staining (n = 5 biological replicates; Scale: 50 μm)

**Supplementary material Fig. 7:**
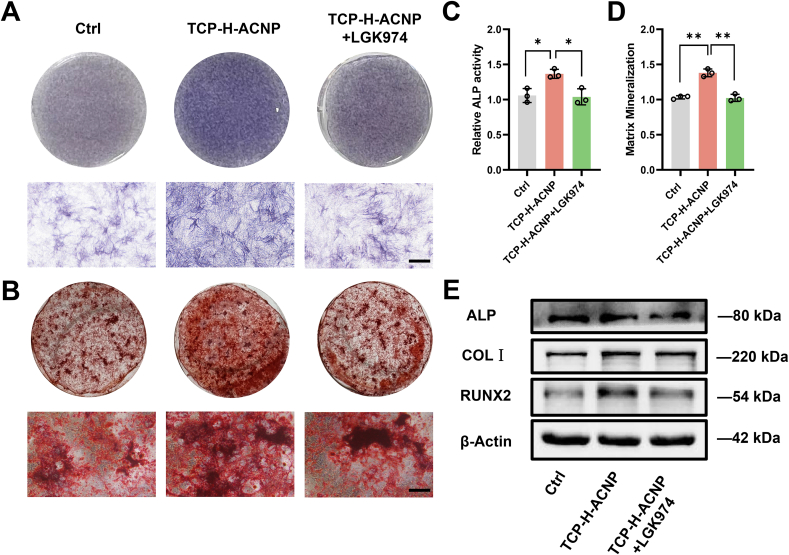
**The TCP-H-ACNP scaffold promotes osteogenic differentiation of OVX-BMSCs through the Wnt signaling pathway. (A)** Histochemical detection of ALP activity in OVX-induced osteoporotic BMSCs following 7-day osteoinductive culture (n = 5 biological replicates; Scale: 100 μm). **(B)** ARS staining after 21-day differentiation protocol (n = 6 independent experiments; Scale: 100 μm). **(C)** Spectrophotometric quantification of ALP enzymatic activity normalized to total cellular protein (n = 3). **(D)** Quantitative mineralization analysis through ARS destaining spectrophotometry (n = 3). **(E)** Western immunoblotting validation of ALP, COL I and RUNX2 protein expression levels (n = 3 biological replicates). Statistical significance thresholds: ∗*p* < 0.05, ∗∗*p* < 0.01 (two-tailed ANOVA with Tukey post hoc).

To elucidate the regulatory mechanism of TCP-H-ACNP hydrogel scaffolds on bone homeostasis, RNA sequencing was conducted. The volcano plot of differentially expressed genes (DEGs) revealed that, compared with the control group, 265 DEGs were upregulated and 156 were downregulated in the TCP-H-ACNP hydrogel scaffold group (Fig. 7A). To elucidate functional differences between experimental groups, we conducted GO enrichment analysis of the sequencing data. The analysis revealed significant upregulation of biological processes related to extracellular matrix organization and calcium ion transport in cells cultured on TCP-H-ACNP hydrogel scaffolds (Fig. 7B). The KEGG enrichment analysis of the signaling pathways indicated that the calcium ion-related signaling pathways presented significant expression differences (Fig. 7C). Through screening by heat maps and verification via RT-qPCR, we discovered that among the components of extracellular matrix composition, *Hmgcs2, Nags, Otc,* and *Pdk4* were significantly upregulated; whereas in the related components of calcium ion transport, genes such as *Adcyap1r1, Atp2a3, Cacna1h,* and *Kcnn4* were significantly upregulated (Fig. 7D–F&G). Meanwhile, we evaluated the mitochondrial function of osteoporotic rat-derived BMSCs. We found that the mitochondrial-related genes *Fis-1, Drp-1,* and *Pgc-1α* were significantly improved in the TCP-H-ACNP group, suggesting that with the enhancement of calcium ion transport signaling pathways, the mitochondrial function of osteoporotic rat-derived BMSCs was improved (Fig. 7E). With the improvement of mitochondrial function, the antioxidant function of osteoporotic rat-derived BMSCs was also significantly enhanced (Fig. 7H). The results of the reactive oxygen (ROS) staining indicated that the TCP-H-ACNP scaffold was able to significantly reverse the trend of increased ROS in osteoporotic rat-derived BMSCs (Supplementary Fig. 6). At the same time, through the display of sequencing results, we found that the Wnt signaling pathway related to osteogenic differentiation was significantly enhanced. Through RT-qPCR verification, we discovered that Wnt4, Wnt5a and Wnt10b played important roles in it. The results of Western blot also showed a consistent trend. We speculated that the TCP-H-ACNP scaffold could also directly promote the osteogenic differentiation of osteoporotic rat-derived BMSCs by activating the Wnt signaling pathway (Fig. 7I and J). To further demonstrate the crucial role of the Wnt signaling pathway in TCP-H-ACNP's promotion of osteogenic differentiation of osteoporotic rat-derived BMSCs, we inhibited the partial function of the Wnt signaling pathway by using the Wnt signaling pathway inhibitor LGK974. We found that after adding LGK974, the effect of TCP-H-ACNP on promoting the expression of ALP in BMSCs was weakened (Supplementary Fig. 7A and C), and the formation of mineralized nodules was also inhibited (Supplementary Fig. 7B and D). The expression of osteogenic-related proteins ALP and RUNX2 was significantly decreased compared to the TCP-H-ACNP intervention group (Supplementary Fig. 7E). This also proved from the opposite side that the Wnt signaling pathway plays a key role in the process of TCP-H-ACNP scaffolds promoting osteogenic differentiation. The above results show that, on the one hand, the TCP-H-ACNP scaffold can improve the mitochondrial function of osteoporotic rat-derived BMSCs by enhancing calcium ion transport, increase local antioxidant capacity, and thereby improve the cellular function of osteoporotic rat-derived BMSCs. On the other hand, the TCP-H-ACNP scaffold can directly promote the osteogenic differentiation of osteoporotic rat-derived BMSCs through the Wnt signaling pathway (Fig. 7K).

### 3.8 The implantation of TCP-H-ACNP hydrogel scaffolds can ameliorate the local blood supply in the rat femoral bone defect models and facilitate bone regeneration

**Fig. 8:**
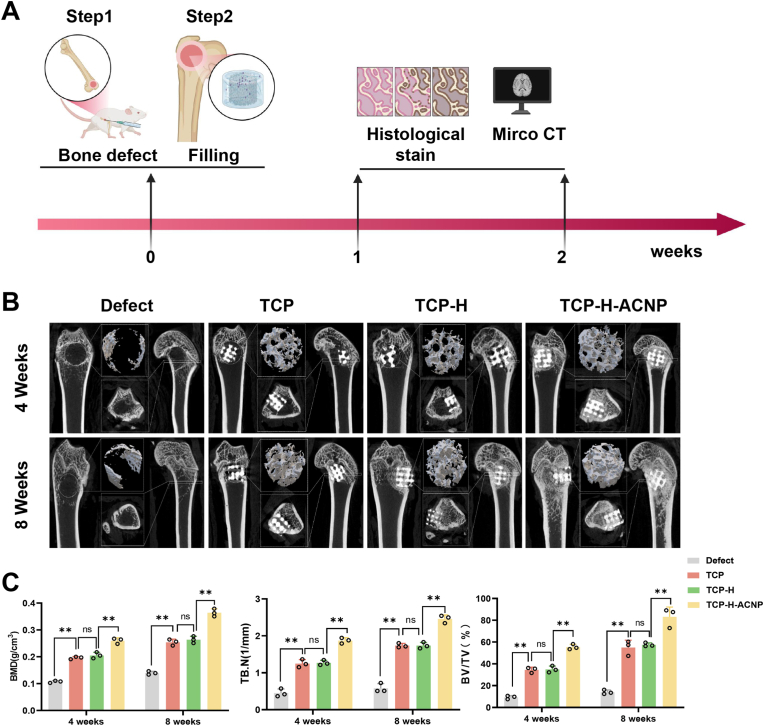
**The *in vivo* bone regeneration of osteoporotic femoral bone defects in rats was assessed at 4 and 8 weeks of treatment. (A)** Schematic illustration of the bone defect model establishment and tissue sampling protocol. **(B)** Micro-CT-based quantitative assessment of osteogenesis, including 3D-reconstructed morphometric analysis of neobone formation in femoral defects. **(C)** Histomorphometric quantification parameters including Bone Mineral Density (BMD), Trabecular Number (Tb.N), and bone volume fraction (BV/TV) were systematically measured in distal femoral specimens (n = 3). Statistical significance thresholds: ∗*p* < 0.05 or ∗∗*p* < 0.01 (two-tailed ANOVA with Tukey post hoc).

Subsequently, we explored the *in vivo* therapeutic effects of TCP-H-ACNP hydrogel scaffolds in osteoporotic rat femoral defect models. The experimental protocol for this part is presented in Fig. 8A. At 4 and 8 weeks post-modeling, rat femoral specimens were harvested. Through two-dimensional and three-dimensional Mirco-CT images, we discovered that, compared with the defect group, the TCP group, the TCP-H group, and the TCP-H-ACNP group all significantly facilitated bone regeneration (Fig. 8B). Quantitative results revealed that, in contrast to the defect group, the TCP group, the TCP-H group, and the TCP-H-ACNP group exhibited significant improvements in bone density (BMD), trabecular number (TB/N), and bone volume fraction (BV/TV) at the defect site, with the TCP-H-ACNP group demonstrating the most pronounced improvement.

**Fig. 9:**
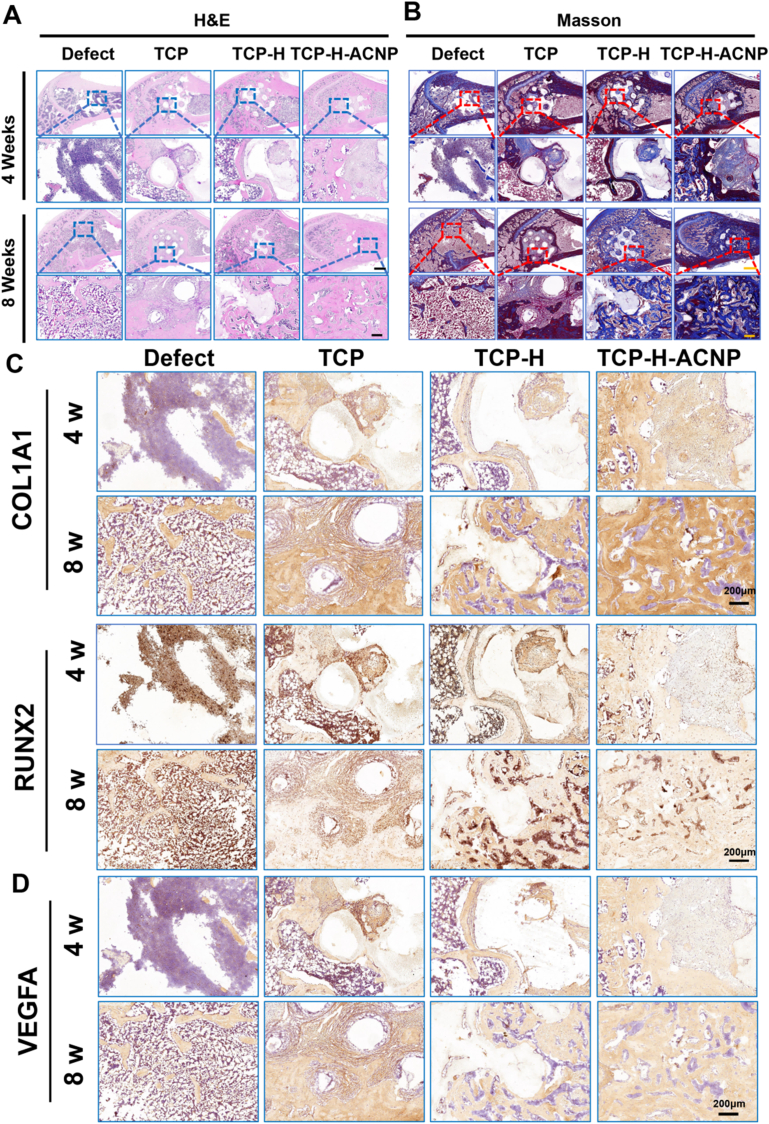
**The *in vivo* bone regeneration of osteoporotic femoral bone defects in rats was assessed at 4 and 8 weeks of treatment. (A)** H&E images of the distal femur after 4 w and 8 w of treatment. **(B)** MTS images of the distal femur after 4 w and 8 w of treatment. **(C)** IHC images of protein COL I and RUNX2. (Scale bar: 100 μm). **(D)** IHC images of VEGF-A. (Scale bar = 100 μm).

**Supplementary material Fig. 8:**
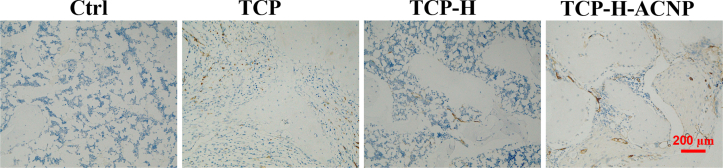
**The TCP-H-ACNP scaffold promotes the expression of the vascular marker CD31 at the site of bone defect.** IHC images of CD31. (Scale bar = 200 μm).

**Supplementary material Fig. 9:**
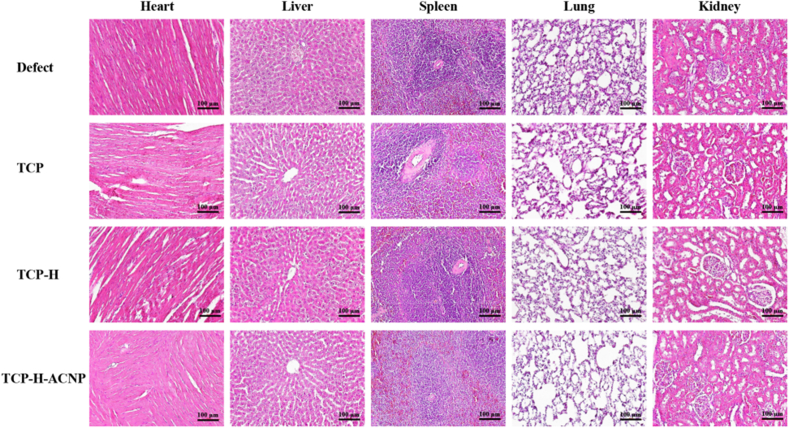
**The long-term Safety of cerium ion accumulation *in vivo*.** The representative H&E images of heart, liver, spleen, lung and kidney tissues, Scale bar = 100 μm.

By assessing the new bone formation using H&E and Masson staining, we found that the TCP-H-ACNP group significantly augmented the formation of new bone (Fig. 9A and B). Immunohistochemical staining of osteogenesis-related proteins indicated that the TCP-H-ACNP group notably increased the expression of type I collagen at the defect site, and the expression of the bone transcription factor RUNX2 was also significantly elevated (Fig. 9C). The results of angiogenesis-related proteins VEGF and CD31 suggested that TCP-H-ACNP was capable of enhancing the local blood supply at the osteoporotic bone defect site. The aforementioned results imply that TCP-H-ACNP hydrogel scaffolds can ameliorate the local blood vessels at the bone defect site in osteoporotic rats and concurrently promote bone regeneration (Fig. 9 D & Supplementary Fig. 8). To ensure the safety of cerium ions in the body, we conducted H&E staining on the heart, liver, spleen, lung and kidney tissues of the rats after 8 weeks of treatment. The results showed that at this concentration, the accumulation of cerium ions in the body did not exhibit significant biological toxicity (Supplementary Fig. 9).

## 4 Discussion

The TCP-H-ACNP scaffold represents a significant advancement in the treatment of osteoporotic bone defects by integrating a hierarchical design approach that combines mechanical support, microenvironmental modulation, and biological regulation. This multi-scale biomimetic system addresses the complex challenges of osteoporosis through a synergistic mechanism involving Wnt signaling activation and mitochondrial function improvement. The scaffold's ability to upregulate Wnt4, Wnt5a, and Wnt10b expression enhances osteogenic differentiation, reversing the Wnt inhibition characteristic of osteoporosis. This is crucial as Wnt signaling plays a pivotal role in bone homeostasis, and its dysregulation is a hallmark of osteoporosis. The scaffold also enhances mitochondrial dynamics by upregulating Adcyap1r1 and Atp2a3, which improves cellular energy metabolism and supports osteoblastogenesis. This dual mechanism addresses the energy deficit in osteoporotic BMSCs, a significant barrier to bone regeneration. There exist numerous regulatory mechanisms governing bone formation. Research on energy metabolism indicates that mitochondria serve as the primary metabolic pathway to meet the ATP requirements during the process of osteoblast differentiation [33].

Recent advances in bone metabolism research have elucidated the involvement of multiple signaling pathways in osteogenesis. The TGF-β/BMP signaling cascade operates through two distinct mechanisms: (1) the canonical Smad-dependent pathway, mediated by TGF-β/BMP ligands, receptors, and downstream Smad proteins, and (2) non-canonical Smad-independent pathways, including the p38 mitogen-activated protein kinase (MAPK) cascade. Upon TGF-β/BMP stimulation, both Smad and p38 MAPK signaling converge on RUNX2, a key transcriptional regulator of mesenchymal progenitor cell differentiation. The synergistic action of TGF-β/BMP-activated RUNX2 and Smads plays a pivotal role in skeletal development [34]. The NF-κB signaling pathway exerts an inhibitory effect on osteoblast dedifferentiation in zebrafish skeletal repair processes [35]. Concurrently, Notch signaling plays a critical role in determining cellular fate, influencing the growth, specialization, and programmed cell death of bone-forming osteoblasts, bone-resorbing osteoclasts, and cartilage-producing chondrocytes [36,37]. Dysregulation of Notch signaling within skeletal tissues—whether through excessive activation or suppression—may contribute to the development of malignant osteosarcoma and age-associated skeletal pathologies, including bone loss (osteoporosis) and joint degeneration (osteoarthritis).

The Wnt signaling pathway serves as a master regulator of osteogenic differentiation. Specifically, the canonical Wnt/β-catenin cascade potently induces stem cell commitment to the osteogenic lineage. This pathway is activated by specific Wnt ligands (Wnt1, Wnt3, Wnt3A, and Wnt10b) that bind to Frizzled (FZD) receptors and LRP5/6 co-receptors, leading to β-catenin stabilization and subsequent transcriptional activation of osteogenic target genes.

The Wnt canonical pathway is of great significance in osteogenic differentiation. Firstly, it can upregulate the expression of the core transcription factors RUNX2 and Osterix, thereby facilitating the differentiation of stem cells into osteoblast [38]. Secondly, it promotes the synthesis of bone matrix and enhances the function of osteoblasts by upregulating markers such as ALP [39], COL1A1 [40], and OCN [41]. In our experimental findings, Wnt10b in the Wnt canonical pathway exhibited a distinct tendency of high expression. Hsu MN et al. reported that the potent and long-term co-activation of Wnt10b and Foxc2 synergistically enhanced the osteogenic differentiation of BMSCs while inhibiting their adipogenic differentiation [42]. Jing H et al. identified a significant reduction in Wnt10b expression levels within BMSCs isolated from osteoporotic murine models. This downregulation was found to directly contribute to pathological bone resorption processes. [43]. These results provide a theoretical foundation for our selection of the Wnt signaling pathway as a major determinant of osteogenic differentiation. Our experimental results further corroborated this. Through transcriptomic analysis and RT-PCR validation, we confirmed that the expression of the Wnt signaling pathway was upregulated following TCP-H-ACNP intervention. Additionally, via inhibitor-based intervention, we demonstrated that the osteogenic differentiation-promoting effect of TCP-H-ACNP through the Wnt canonical pathway was inhibited.

The Wnt signaling pathway regulates the downstream calcium ion transport signaling pathway. The non-canonical Wnt signaling pathways (Non-canonical Wnt pathways) do not depend on the stabilization and nuclear translocation of β-catenin. Instead, they exert their functions by regulating cell polarity, cell migration, calcium signaling, and cytoskeletal reorganization. The main ligands of these pathways include Wnt4, Wnt5a, and Wnt11. Wang et al. demonstrated that stimulation of the Wnt5a signaling cascade facilitates calcium channel activation, thereby promoting myogenic differentiation in C2C12 cells and enhancing the regeneration of injured skeletal muscle tissue [44]. Wan et al. demonstrated that ginsenoside administration induces TBK1-AMPK pathway activation, which enhances omentin production in adipocytes. This effect subsequently stimulates myocardial mitochondrial biogenesis in heart failure via the Wnt5a/calcium signaling cascade [45].

In the results of our experiment, the validation of transcriptome sequencing, RT-PCR, and WB indicated that in osteoporotic rat-derived BMSCs treated with TCP-H-ACNP, the expressions of Wnt4 and Wnt5a were significantly upregulated, and the expressions of genes related to calcium ion transport were also increased. Meanwhile, when evaluating mitochondrial function, it was found that the TCP-H-ACNP hydrogel scaffold could significantly decrease the expression level of ROS in osteoporotic rat-derived BMSCs and enhance the expressions of antioxidant enzymes.

Currently, clinical intervention and treatment options for bone defects are still relatively limited. Traditional autologous and allogeneic bone transplantation and distraction osteogenesis not only have low treatment efficiency and poor efficacy, but also pose a threat to the health of patients during the long recovery process due to the compression and occupation of surrounding tissues and the most common infection. In recent years, the membrane-induced method, which promotes osteogenesis by applying periosteum on both sides of the fracture end, has emerged. Although it can effectively enhance bone conductivity, it often leads to core necrosis and implant failure due to poor mechanical properties and inadequate vascularization. On the other hand, scaffold implantation, with its high mechanical strength and the ability to promote bone and surrounding vascular formation, has gradually gained popularity. Currently, the generally recognized bone substitute materials should meet the "4F" principle: 1. Form: The material should have good plasticity to fill bone defects of different shapes. 2. Function: The scaffold should have good mechanical strength to withstand forces in multiple directions of the limb and partially replace the limb's movement function. 3. Fixation: The scaffold should have good stability and gradually degrade to be replaced by new bone during the bone repair process. 4. Formation: This is also a very important point. Good osteoinductivity, the scaffold implanted in the body needs to have certain bone conduction, bone formation, vascular formation and osteoinductive capabilities to enhance local blood supply and promote bone formation. Compared with these traditional treatment methods, the TCP-H-ACNP scaffold demonstrates superior osteogenesis and angiogenesis capabilities. However, in terms of mechanical support, large animal experiments need to be included to better simulate the physiological load of the human body.

In comparison to existing bisphosphonate therapies, such as alendronate, the TCP-H-ACNP scaffold offers a more comprehensive approach. While alendronate inhibits osteoclast activity, it lacks the ability to directly promote osteogenesis and angiogenesis [[46], [47], [48]]. The incorporation of cerium ions into the scaffold addresses this limitation by enhancing angiogenic and osteogenic activities [[49], [50], [51]]. Cerium's ability to mimic endogenous antioxidant enzymes mitigates oxidative stress, which exacerbates bone loss in osteoporosis [52,53]. The scaffold's angiogenic effects are also noteworthy, as the cerium component enhances HUVEC migration and tubule formation via VEGF upregulation, addressing the vascular insufficiency characteristic of osteoporotic defects [54]. This dual osteogenic-angiogenic effect is critical for osteoporotic bone regeneration, as angiogenesis is essential for delivering nutrients and oxygen to the healing site [55,56].

The scaffold's hierarchical design offers several advantages over single-component materials. The 3D-printed TCP framework provides mechanical support [57], while the COMA hydrogel mimics the organic ECM, creating a hydrated environment conducive to cell adhesion and differentiation [58]. The integration of ACNP particles introduces targeted bioactivity, ensuring sustained release and localized therapeutic effects. This approach aligns with emerging trends in ion-doped biomaterials, aiming to enhance the biological performance of bone substitutes. The scaffold's ability to enhance bone regeneration and mechanical properties positions it as a promising candidate for clinical application.

In terms of Clinical transformation, the TCP-H-ACNP scaffold demonstrates superior performance compared to conventional materials. Comparative analysis reveals that the scaffold significantly outperforms commercial β-TCP and HA/collagen scaffolds in terms of bone volume fraction and bone mineral density [59,60]. This suggests that the scaffold's multi-scale design and dual therapeutic ions provide a more effective solution for osteoporotic bone defects. However, several challenges remain for clinical translation, including long-term safety, scalability, and stability. The potential for cerium accumulation must be thoroughly evaluated, and the scalability of the 3D-printed TCP framework and the stability of the hydrogel need to be optimized for large-scale production. Further studies are also required to assess the scaffold's immunomodulatory effects, as the modulation of macrophage polarization could significantly enhance the regenerative microenvironment.

The TCP-H-ACNP scaffold's hierarchical design and dual therapeutic ions represent a significant departure from traditional bone graft substitutes, offering a more effective solution for osteoporotic bone regeneration. The scaffold's ability to modulate both osteogenic and angiogenic processes provides a comprehensive approach to bone healing. The integration of cerium ions not only enhances bone formation but also mitigates oxidative stress, a key factor in osteoporosis progression. This dual functionality sets the TCP-H-ACNP scaffold apart from other ion-doped materials, which often focus on a single aspect of bone regeneration. Moreover, the scaffold's hierarchical structure mimics the natural bone architecture, providing both mechanical support and a conducive environment for cell adhesion and proliferation. This design ensures that the scaffold can integrate well with the host tissue, promoting long-term stability and functionality. The combination of mechanical robustness and biological activity makes the TCP-H-ACNP scaffold a promising candidate for clinical applications in osteoporotic bone repair.

The compressive strength requirement for cancellous bone substitute materials is approximately 2–50 MPa. Under this size condition, our TCP scaffold can withstand a compressive strength of (8.683 ± 0.413) MPa, which meets the requirements of cancellous bone substitute materials. However, the 3D printed TCP scaffold under this size condition can only be applied to the rat femoral bone defect model. Due to the absence of the Haversian system, the bone remodeling in rats is mainly primary bone, which cannot simulate the complex secondary bone reconstruction process in humans. In addition, the rat bone defect model cannot replicate the physiological mechanical load of humans in terms of stress loading, which also makes it difficult to truly reflect the transformation performance of the TCP-H-ACNP scaffold in this model. This is also the limitation of our experiment.

Therefore, further evaluation of the translational potential on large animal bone defect models is part of our future experimental plan. Firstly, in terms of animal selection, the anatomy and physiology of the femur in pigs are similar to those in humans, making them a suitable choice for long bone defect models. Shuai et al. used Bama pigs to evaluate the therapeutic effect of scaffolds on large segmental bone defects [61]. Meanwhile, although the 3D printed TCP scaffolds with a diameter of 3 mm can meet the mechanical requirements of bone defects in rats, they fail to do so in large animal models. Therefore, it is necessary to improve the size of the 3D printed TCP scaffolds.

In the context of translational research, several key factors, namely large-scale production, cost control, and clinical operability, significantly influence the successful translation of our research results. The inherent layer-by-layer manufacturing process of 3D printing poses challenges to the large-scale and efficient production of TCP scaffolds. This must be carefully considered as it directly affects the scalability of the solutions we propose. Additionally, the high cost of medical-grade tricalcium phosphate is a major limitation to its widespread application. Therefore, developing strategies to reduce the cost of tricalcium phosphate while maintaining its quality and performance is a key consideration for future experimental efforts. Improving cost-effectiveness is crucial for the wider application of this material in clinical settings. Clinical operability is a key determinant of the translational potential of scaffolds. The combination of CT-based 3D reconstruction and 3D printing technology offers a promising solution. Through digital imaging techniques, the three-dimensional anatomical structure of the bone defect site can be precisely captured [62]. This enables the manufacture of customized 3D printed scaffolds that perfectly fit the defect area, facilitating surgical implantation and significantly reducing the complexity of clinical operations.

## 5 Conclusion

The TCP-H-ACNP scaffold represents a significant step forward in the treatment of osteoporotic bone defects. Its hierarchical design, synergistic mechanisms, and superior performance compared to existing materials position it as a promising candidate for clinical application. The TCP-H-ACNP scaffold exhibits enhanced mechanical properties, more accurate microenvironment mimicry, and robust bioactive functions. Future research will focus on addressing the remaining challenges to advance this technology towards clinical translation, ultimately providing a comprehensive solution for osteoporotic bone regeneration. The scaffold's ability to modulate both osteogenic and angiogenic processes provides a comprehensive approach to bone healing. The integration of cerium ions not only enhances bone formation but also mitigates oxidative stress, a key factor in osteoporosis progression. This dual functionality sets the TCP-H-ACNP scaffold apart from other ion-doped materials, which often focus on a single aspect of bone regeneration. Moreover, the scaffold's hierarchical structure mimics the natural bone architecture, providing both mechanical support and a conducive environment for cell adhesion and proliferation. This design ensures that the scaffold can integrate well with the host tissue, promoting long-term stability and functionality. The combination of mechanical robustness and biological activity makes the TCP-H-ACNP scaffold a promising candidate for clinical applications in osteoporotic bone repair. However, the successful translation of this technology to the clinic requires careful consideration of several factors. Long-term safety assessments are essential, particularly regarding the potential for cerium accumulation in the body. The scaffold's scalability and stability must also be optimized to ensure consistent performance across different batches and manufacturing scales. Additionally, understanding the scaffold's immunomodulatory effects is crucial, as the modulation of immune responses can significantly influence the regenerative process. In conclusion, the TCP-H-ACNP scaffold represents a significant step forward in the treatment of osteoporotic bone defects. Its hierarchical design, synergistic mechanisms, and superior performance compared to existing materials position it as a promising candidate for clinical application. Future research will focus on addressing the remaining challenges to advance this technology towards clinical translation, ultimately providing a comprehensive solution for osteoporotic bone regeneration.

## Authorship

Conception and design of study: Yesheng Jin, Yong Xu, Fan He, Gang Zhao; acquisition of data: Yesheng Jin, Shuqing Lv, Nanning Lv, Yixue Huang; analysis and/or interpretation of data: Yesheng Jin, Shuqing Lv, Nanning Lv, Yixue Huang, Jia Wang, Yun Xiao, Xinfeng Zhou, Yanxia Ma; Drafting the manuscript: Yesheng Jin; revising the manuscript critically for important intellectual content: Fan He, Gang Zhao, Yong Xu. Approval of the version of the manuscript to be published (the names of all authors must be listed): Yesheng Jin, Shuqing Lv, Nanning Lv, Yixue Huang, Jia Wang, Yun Xiao, Xinfeng Zhou, Yanxia Ma, Gang Zhao, Fan He, Yong Xu.

## Conflicts of interest

The authors have declared that no competing interest exists.
