# Supplementary material for: Multi-scale biomimetic fusion construction of cerium ion hydrogel-scaffold for promoting osteoporotic bone defect repair
Source: J Orthop Translat. 2025 Sep 13;55:172–91. doi: 10.1016/j.jot.2025.08.015 (PMC12799519; doi:10.1016/j.jot.2025.08.015)
Supplement: Multimedia component 1 [file mmc1.docx]

**Table1 Sequences of Rat primers for the qRT-PCR analysis**

| **Gene** | **Primer forward（5’-3’）** | **Primer reverse（3’-5’）** |
| --- | --- | --- |
| **Adcyap1r1** | CTGCATCTTCAAGAAGGAGC | CACAAGCATCGAAGTAGTGG |
| **Atp2a3** | CCTGCTCTCGGCTGCCGACG | GTGGACTTGATCTCGATGAG |
| **Cacna1h** | GGCGAAGAAGGCAAAGCTGA | GCGTGACACTGGGCATGTT |
| **Kcnn4** | GCTGTTCATGACTGACAACG | CATAGCCAATGGTCAGGAAC |
| **Pdk4** | AATCAAGATTTCTGACCGAG | CTGACATGGAATAGAGATTCAG |
| **Otc** | GGGAAGCCAGTCCAGAGTCA | CCTCCCAGAAGAGCGAAGCC |
| **Wnt4** | GCCACGCACTAAAGGAGAAG | TCATCCGTATGTGGCTTGAA |
| **Wnt5a** | AGCCGAGAGACAGCCTTCAC | TCCTGCGACCTGCTTCATTG |
| **Wnt10b** | GTGGGGAAACTGTGTGGAGT | CCGTGAGTTAGGTCGAGCAG |
| **Mfn1** | CCATCACTGCGATCTTCGGCCA | CAGCGAGCTTGTTTCTGTAGCCCT |
| **Fis1** | CGTGCTTTCTGTAACGCCTG | CTACAGGCACTTTGGGGGTT |
| **Drp1** | GAGAACTACCTTCCGCTGTATCGC | CACCATCTCCAATTCCACCACCTG |
| **Sod-2** | GCTTGATAGCCTCCAGCAAC | GGCCAAGGGAGATGTTACAA |
| **Cat** | ACATGGTCTGGGACTTCTGG | CAAGTTTTTGATGCCCCTGGT |
| **Nrf-2** | GCTATTTTCCATTCCCGAGTTAC | ATTGCTGTCCATCTCTGTCAG |
| **Gapdh** | GCAAGTTCAACGGCACAG | CGCCAGTAGACTCCACGAC |
